# Limited Plasticity of Stomatal Development in Apple Trees Across Diverse European Climates

**DOI:** 10.1111/ppl.71000

**Published:** 2026-07-05

**Authors:** Francesca Zuffa, Michaela Jung, Steven Yates, Carles Quesada‐Traver, Maria José Aranzana, Lidia Lozano, Elias Holzknecht, Walter Guerra, François Laurens, Hélène Muranty, Andrea Patocchi, Bruno Studer, Graham Dow

**Affiliations:** ^1^ Molecular Plant Breeding, Institute of Agricultural Sciences ETH Zurich Zurich Switzerland; ^2^ Plant Breeding Division Agroscope Waedenswil Switzerland; ^3^ IRTA (Institut de Recerca i Tecnologia Agroalimentàries) Barcelona Spain; ^4^ Centre for Research in Agricultural Genomics (CRAG) CSIC‐IRTA‐UAB‐UB Barcelona Spain; ^5^ Laimburg Research Center Pfatten Italy; ^6^ Univ Angers Institut Agro, INRAE, IRHS, SFR QUASAV Angers France; ^7^ Crop Science and Production Systems NIAB Cambridge UK

**Keywords:** acclimation, breeding, *Malus domestica*, plasticity, resilience, stomata

## Abstract

Climate change affects plant acclimation and adaptation, particularly in long‐lived perennial crops like apple ( *Malus domestica* Borkh.). Stomata play an essential role in regulating these plant environmental responses. This study examined variation in stomatal density (SD) and stomatal function of apple trees in 2022–2023 across four European locations (Spain, ESP; France, FRA; Italy, ITA; and Switzerland, CHE, the only non‐irrigated orchard). A total of 20 preselected apple accessions with extreme SD phenotypes (HSD and LSD) and five commercial varieties (MSD‐Comm) were compared. Across locations, SD was consistent for each SD group, but partial plasticity emerged under heat stress in ESP and FRA. In ESP, SD increased under high temperatures and irrigation, but the increase was only significant for MSD‐Comm (*p* < 0.05). In FRA, HSD decreased under high temperatures and limited irrigation in 2022, but these changes reversed under wetter conditions in 2023 (*p* < 0.05), reinforcing SD stability and the capacity for local acclimation. Net carbon assimilation (*A*_*net*_) showed no significant differences across SD groups and locations. Stomatal conductance (*g*_*s*_) adjusted to local climate and produced variations in intrinsic water‐use efficiency (iWUE). Conditions in FRA, ITA, and CHE also led to a significant correlation between integrated WUE (δ^13^C) and Total Fruit Weight (*p* < 0.01, *R* ^*2*^ = 0.20). In contrast, high temperatures and irrigation in ESP maintained no correlation between δ^13^C and Total Fruit Weight. These results highlight the role of SD in short‐term acclimation and, given its stability across environments, great potential for breeding climate‐adapted apples.

## 1 Introduction

Climate change predictions indicate a rise in global temperatures, atmospheric vapor pressure deficit (VPD) and extended periods of droughts (IPCC 2022). These abiotic factors trigger physiological responses in plants, shaping their climate adaptation strategies (Novick et al. 2024). Plants possess the ability to respond to short‐term environmental changes, which can be perceived as stress, through acclimation (Filippou et al. 2013; Allen et al. 2015). Unlike genetic adaptation, acclimation involves phenotypic modifications within an individual plant's lifespan (Lee et al. 2024). The changes in anatomical and physiological traits during leaf emergence and expansion refer to developmental acclimation, whereas dynamic acclimation is associated with adjustments after leaf development has been completed (Zhang et al. 2024). These modifications provide an immediate plastic response to environmental stress and potentially influence long‐term climate adaptation that enables plants to maintain optimal function in a changing environment (Violle et al. 2007; Jump et al. 2009). Acclimation mechanisms are often mediated through functional traits, which represent quantitative links between optimal plant performance and environmental conditions (Reich et al. 2003; Nicotra et al. 2010; Bertolino et al. 2019; Campany et al. 2019). Stomatal traits have traditionally been linked to environmental acclimation and represent potential targets to improve abiotic stress resilience in crops (Bertolino et al. 2019; Leakey et al. 2019; Gray and Dunn 2024).

Stomata are microscopic pores on aerial plant surfaces that regulate gas exchange, allowing carbon dioxide uptake for photosynthesis while controlling water loss via transpiration (Dow and Bergmann 2014; Lawson and Vialet‐Chabrand 2019). The variability of stomatal traits across different environmental conditions reflects the important trade‐offs between carbon acquisition and water conservation, enabling plants to efficiently withstand a wide range of environmental conditions (Hetherington and Woodward 2003; Bertolino et al. 2019; Lawson and Vialet‐Chabrand 2019). This trade‐off is especially important under drought stress, when plants exhibit a spectrum of stomatal regulation strategies to sustain hydraulic function (Brodribb and Feild 2000; Sade and Moshelion 2014). Perennial plants, such as trees, endure a greater range of environmental conditions over the years and show substantial phenotypic plasticity and genetic adaptation in their reproductive traits, growth patterns and resource allocation (Atlan et al. 2015; Cortés et al. 2020). Specifically, stomatal density (SD) is known to co‐vary with environmental gradients, often increasing with growing season temperature, aridity and latitude (Reich et al. 2003; De Carcer et al. 2017; Liu et al. 2024). Plants in drier environments typically exhibit smaller, more densely distributed stomata that provide higher leaf transpirational cooling (Iqbal et al. 2020). Inversely, plants in more humid regions often have fewer, larger stomata, minimizing the metabolic costs of stomatal production to increase their maximum photosynthetic returns (Fanourakis et al. 2020). These previous studies of stomatal development highlight the environmental plasticity of these traits (Klein et al. 2013; Reddy et al. 2019). In this context, stomatal traits also influence plant survival and reproduction, suggesting that adjusting SD might be a potential long‐term strategy for breeding plants that can adapt to future environmental conditions (Bucher et al. 2017). Given prior studies demonstrating that SD can vary in response to environmental conditions, SD in apple might also exhibit environmental‐based variation. However, it remains unclear to what extent variation may be affected by genotype‐specific differences in SD, particularly given that apple trees in a singular location maintain consistent SD across years (Zuffa et al. 2024). This consistency suggests strong genetic control or potential acclimation to local environments over longer time periods (Zuffa et al. 2024). Despite the evidence of these patterns, the role of stomatal development in driving crop productivity across diverse environments remains underexplored, especially for perennial crops like apple.

Apple ( *Malus domestica* Borkh.) is an excellent model for studying stomatal trait variation due to the widespread cultivation across diverse climates globally, from temperate to semi‐arid regions, where irrigation is vital to maintain productivity (Naor 2006; Naor et al. 2008; FAO 2021). Additionally, apple is one of the world's most economically important fruit crops (FAO 2021), and understanding how apple trees acclimate to different environments may have implications for improving crop productivity and climate resilience (Ramirez and Kallarackal 2015). Maintaining apple production under stressful environmental conditions presents a dual challenge: producers must adjust management practices to meet the physiological demands of apple trees (Jin et al. 2022), while breeders need to identify and incorporate key traits into future apple cultivars (Karlström et al. 2019). Stomatal traits, with their direct link to water‐use efficiency and photosynthesis, are prime candidates for breeding (Bertolino et al. 2019; Leakey et al. 2019; O'Rourke 2021; Gray and Dunn 2024). Investigating stomatal plasticity across different locations can offer valuable insights into their functional significance and their potential roles in tree crop acclimation (Gratani 2014). By integrating stomatal function and environmental acclimation into plant breeding strategies, production can better meet the challenges of climate change while ensuring sustainable cultivation practices (Leakey et al. 2019).

The objectives of this study were: (i) to investigate the phenotypic variation in SD among diverse apple accessions grown in different locations; (ii) to determine whether variation in SD is associated with physiological differences in leaf gas exchange and the climatic conditions of the growth location; and (iii) to assess SD influences on apple fruit production and tree resilience across different locations. We hypothesize SD will vary across locations in response to local environments and this variation will drive changes in leaf gas exchange and tree productivity.

## 2 Material and Methods

### 2.1 Plant Material and Location Description

The plant material was comprised of the apple REFPOP accession group, as previously described by Jung et al. (2020). Briefly, this material consisted of 269 accessions representing a population of wide‐ranging apple genetic diversity, whereby each accession was planted once in two separate randomized complete blocks. At each location, an additional two incomplete randomized blocks may have contained a further two trees per accession (maximum of four trees per accession per location). Three control accessions: ‘Gala’, ‘Golden Delicious’, and ‘CIVG198’, known as ‘Modì’, were replicated up to 22 times at each location. Primary fruits were hand‐thinned after the June fruit drop and up to two apples per fruit cluster were retained. The apple REFPOP followed a multi‐location design, and this study focused on four apple REFPOP locations: (i) Angers, France (FRA) (47.4842° N, −0.6135° E), (ii) Lleida, Spain (ESP) (41.6727° N, 0.4020° E), (iii) Laimburg, Italy (ITA) (46.3442° N, 11.2781° E), and (iv) Waedenswil, Switzerland (CHE) (47.2211° N, 8.6669° E). Orchard‐specific management was employed based on the practices of each country, incorporating integrated plant protection methods. Unlike the orchards in ESP, FRA, and ITA, the orchard in CHE was not irrigated. Weather data was collected from weather stations installed near the apple REFPOP orchards at each location (Figure S1). To characterize the climate of each location, the mean maximum daily temperature and vapor pressure deficit (VPD) were calculated by extracting the daily maximum values of temperature and VPD, respectively, and averaging these values between 12:00 and 14:00 across 6 months (April, May, June, July, August, September). Furthermore, the Palmer Drought Severity Index (PDSI) was calculated as a measure of the drought intensity and extent (Alley 1984). Irrigation data (Figure S1) were included in the water balance estimation used to calculate PDSI. Potential evapotranspiration was estimated with the radiation‐based Hargraves equation (Hargreaves and Allen 2003):

(1) $${\mathrm{PE}}_{T}=0.0135\times \left(T_{\mathrm{avg}}+17.78\right)\times R_{a}\times \left(\frac{238.8}{\left(595.5-0.55\times T_{\mathrm{avg}}\right)}\right)$$

where ${\mathrm{PE}}_{T}$ is the potential evapotranspiration, $T_{\mathrm{avg}}$ is the average temperature, $R_{a}$ is the solar radiation.

### 2.2 Stomatal Density Quantification and Analysis

A total of 10 accessions representing the upper 4% (high stomatal density, HSD) and 10 accessions representing the lower 4% (low stomatal density, LSD) of the population‐wide stomatal density (SD) distribution were selected from the apple REFPOP as previously described by Zuffa et al. (2024) (list of accessions in Table S1). Additionally, five commercial genotypes of the apple REFPOP (‘Gala’, ‘Golden Delicious’, ‘CIVG198’, ‘McIntosh’ and ‘Jonathan’), falling within the middle range of the SD distribution (MSD), were also included. These five accessions were collectively identified as “MSD‐Comm”.

From two trees of each HSD, MSD‐Comm and LSD accession, six sun‐exposed, fully mature, and healthy leaves from different primary branches were collected in summer 2022 in ESP, FRA, ITA, and CHE, and in 2023 in ESP and FRA. SD quantification and analysis were performed as previously described by Zuffa et al. (2024). Briefly, to prepare stomatal imprints, two successive layers of clear nail polish (Wild Shine Nail Color, Protective Base Coat, Wet n Wild) were applied to the abaxial leaf surface between major veins in the mid‐region of the leaves. After drying, each layer was carefully removed using Eco&Clear tape (Tesa SE): the first layer to eliminate leaf trichomes and the second layer to create clear leaf imprints.

SD images (872 × 654 μm) were captured using bright‐field microscopy (DM 2500 upright microscope with a DFC 7000 T camera, Leica Microsystems) at 10× magnification. To capture leaf variability, images were taken at three non‐adjacent locations on each imprint (top, middle, and bottom), resulting in three images per leaf. A total of 6268 images across all locations and years (2022–2023) were obtained. An automated stomatal density counting model was applied to all images for consistent SD measurements (Zuffa et al. 2024). Following SD analysis, these leaves were maintained for additional leaf trait and elemental tissue analyses.

### 2.3 Leaf Traits and Elemental Tissue Analysis

In 2022 and 2023, leaves from HSD, LSD, and MSD‐Comm accessions were imaged with a flatbed scanner (Canon Scan Lide 400, Canon Inc.). Leaf area (LA, cm^2^) was measured manually from these images using ImageJ software. After imaging, leaves were oven‐dried at 60°C for 72 h. Prior to drying, a punch with a 2 cm diameter was taken from each leaf (between the central vein and the edge) for leaf mass per area (LMA, g cm^−2^) and elemental tissue analysis. The trait LMA was calculated as the ratio of dry leaf mass to punch diameter for each sample. In 2022, three leaf discs per replicate tree were ground (Mixer Mill MM 200, Retsch), and processed to create three distinct biological samples per tree. Carbon and nitrogen isotope discrimination (δ^13^C, δ^15^N) and elemental composition (%C, %N) analyses were conducted on ~3.6 mg of ground leaf tissue contained in 5 × 9 mm tin capsules (Saentis Analytical AG). Samples were combusted in a Flash EA 1112 series elemental analyzer (Thermo Italy, former CE Instruments), linked to a Finnigan MAT DeltaplusXP isotope ratio mass spectrometer via a 6‐port valve (Brooks et al. 2003) and a ConFlo III interface (Finnigan MAT) following Werner et al. (1999). To ensure accuracy, the samples, blanks, and standards were prepared according to the Identical‐Treatment principle (Werner and Brand 2001) with results adjusted for blanks. Quality control standards indicated an uncertainty of 0.2‰ for both δ^13^C and δ^15^N measurements, 7.7% ± 0.3% for nitrogen content (using tyrosine), and 4.4% ± 0.4% for carbon content (using caffeine). Carbon isotope discrimination was used as a proxy for integrated water‐use efficiency (both denoted as δ^13^C), and reflected the ratio of ^13^CO_2_ to ^12^CO_2_ in leaf dry matter (Franks and Farquhar 2007).

### 2.4 Leaf Gas‐Exchange Measurements

Net carbon assimilation (*A*_*net*_, μmol CO_2_ m^−2^ s^−1^) and stomatal conductance (*g*_*s*_, mol H_2_O m^−2^ s^−1^) were measured following the protocol established by Zuffa et al. (2024). Intrinsic water‐use efficiency (iWUE) was determined as the ratio between *A*_net_ and *g*_s_. In brief, the measurements were taken between 08:30 and 13:00, depending on the specific daily weather conditions, using a portable infrared gas exchange system (LI‐6800 with LED light source and a 6 cm^2^ chamber, LI‐COR Biosciences). The timeframe between 8:30 and 13:00 was necessary to fit the repeated sampling of trees within the fieldwork time period. In addition, different genotypes and tree replicates were sampled on different days and at different times of morning to minimize potential sampling bias. Instantaneous measurements (lasting 1 to 3 min, depending on leaf stabilization) were obtained from the central lamina of three sun‐exposed leaves from two tree replicates for each of the HSD, LSD, and MSD‐Comm accessions. These leaves were sampled at mid‐height on separate, south‐facing vegetative primary branches. Mature leaves were classified as mid‐branch, at least 8 leaves away from the branch tip. The sampling was performed across all accessions and trees from late June to late August in 2022 and repeated twice at each location (in ESP, FRA, ITA, and CHE) and in 2023 (in ESP and FRA), with two sampling weeks per year and location. Measurement parameters included flow rate 600 μmol s^−1^, fan speed 10,000 rpm, [CO_2_] 415 μmol mol^−1^, and photosynthetically active radiation (PAR) at 1500 μmol photons m^−2^ s^−1^. Leaf temperature (T_leaf_, °C) and relative humidity (RH, %) were not directly controlled in the chamber to avoid long stabilization times; however, RH was adjusted to fall between 50% and 80% if ambient RH fell outside of this range. Preliminary measurements on the control accessions ‘Golden Delicious’, ‘Gala’, and ‘CIVG198’ (‘Modì’) in the apple REFPOP in CHE showed that branch type and canopy position had no effect on gas exchange in sun‐exposed leaves (Zuffa et al. 2024). In contrast, trees without fruit exhibited markedly lower gas exchange, with stomatal conductance (*g*_*s*_) approximately 40% lower (Zuffa et al. 2024). Therefore, all physiological analyses were conducted exclusively on fruit‐bearing trees.

### 2.5 Stomatal Response Curves to Changing Light Across Locations

In 2022, one random accession from each SD group (‘Rouget’ for LSD, ‘Priscilla’ for HSD, and ‘Gala’ as the MSD‐Comm) was chosen at all locations to evaluate stomatal response curves. Curve replicates were done on different trees from the same accession. For each accession, three response curves were conducted under varying light intensities controlled by the LI‐6800 light source: 1500 PAR, 100 PAR, and back to 1500 PAR. Gas‐exchange measurements were recorded every minute throughout the entire response curve. Leaves were first stabilized at 1500 PAR (typically taking 15–30 min) before data collection began, logging for 5 min at this light level. The light intensity was then reduced to 100 PAR for a 30‐min response phase and then increased back to 1500 PAR for an additional 30‐min recovery phase. Although gas exchange did not always reach a fully stable steady state within 30 min after the light transitions, waiting until a complete steady state was reached under open‐field conditions was not feasible due to the limited sampling window. Therefore, we standardized the duration of both phases to a fixed time frame to ensure comparability across genotypes and light treatments and to evaluate their responses within a consistent and practical measurement period. Environmental conditions inside the chamber were maintained at a flow rate of 600 μmol s^−1^; fan speed 10,000 rpm; [CO_2_] 415 μmol mol^−1^. RH and T_leaf_ were set according to the ambient environmental conditions but always maintained within the range of 50%–60% and 28°C–34°C, respectively. To evaluate stomatal kinetics independently of the initial and absolute changes in *g*_s_, relative *g*_s_ values were calculated by normalizing *g*_s_ to the maximum value in each curve. The stomatal response to a decrease in light intensity to 100 PAR was modeled using an exponential function (Equation 2), while the recovery phase (light increase to 1500 PAR) was fitted with a sigmoid function (Equation 3) following Vialet‐Chabrand et al. (2017). The exponential equation to describe the decrease in *g*_s_ was:

(2) $$g_{s}=G_{\min}+\left(G_{\max}-G_{\min}\right)e^{-t/\tau_{d}}$$

where *t* is the time since the change in PAR, $G_{\min}$ and $G_{\max}$ indicate the minimum and maximum steady‐state *g*_s_, and $\tau_{d}$ represents the time constant defining the time required to reach 63% of the total variation when $\tau_{d}$ = t, $\frac{g_{s}-G_{\min}}{G_{\max}-G_{\min}}=1-e^{-1}\sim 0.63).$ The sigmoid function that describes the increase in *g*_s_ after a stepwise increase in light was:

(3) $$g_{s}=\left(G_{\max}-G_{\min}\right)\;e^{-e^{\left(\frac{\lambda \;-t}{k_{i}}+1\right)}}+G_{\min}$$

where $k_{i}$ is the time constant for the initial increase of *g*_s_ and λ is the initial lag time, defined here as the time taken for *g*_s_ to reach the 10% above the minimum value $G_{\min}$.

Additional parameter $\tau_{i}$, the time constant required to reach 63% of the total variation, was obtained for each replicate measurement in the recovery phase as:

(4) $$\tau_{i}=\lambda -k_{i}\cdot \left[\ln\left(-\ln\left(1-e^{-1}\right)\right)-1\right]$$

To describe the speed of stomatal response, maximum slope of *g*_s_ increase (${\mathrm{Sl}}_{\max}$) was calculated as:

(5) $${\mathrm{Sl}}_{\max}=k_{i}\cdot \frac{G_{\max}-G_{\min}}{e}$$

For *A*_net_, the parameter $\tau_{a}$ was obtained for each replicate measurement across the response and the recovery phase, with $\tau_{a}$ representing the time required to reach 95% of the total *A*_net_ variation (McAusland et al. 2016).

### 2.6 Productivity Traits

The traits Total Fruit Weight (TFW) and Trunk Diameter (TD) were collected in 2022 as described by Jung et al. (2022). TFW, measured in kilograms, was determined using either scales in ESP, FRA and ITA, or a sorting machine (Greefa iQS4 v.1.0) in CHE. Measurements were taken from the entire fruit production or a specific batch. When a batch was weighed, TFW was estimated by multiplying the average fruit weight—calculated from the batch—by the total number of fruits produced. Prior to flowering, TD (mm) was measured 20 cm above the grafting point using a digital caliper. At ITA, the trunk circumference was recorded instead and converted afterward to diameter using the standard circumference‐to‐diameter ratio.

### 2.7 Statistical Data Analysis

Statistical analyses were performed using R (v.4.4.2, R Development Core Team 2010), and figures were generated with the package ggplot2 (v.4.2.1). The traits for each SD group were categorized into three main groups: stomatal traits (SD, *g*_*s*_, *A*_*net*_, iWUE), leaf traits [leaf area (LA), LMA, N_a_, leaf carbon content (C%), N%, δ^13^C, δ^15^N], and productivity traits (TFW, Trunk Diameter). Stomatal, leaf and productivity traits were all collected from the same trees, allowing for paired data analysis. Therefore, pairwise comparisons of stomatal traits, leaf traits, and TFW across different SD groups and locations were done using Wilcoxon tests (‘wilcox_test’ function from the rstatix package, v.0.7.2) and *p*‐values adjusted with the Bonferroni correction. Trees without fruit in either of the two studied years at each location were excluded from these comparisons, as crop load status significantly influences tree physiological activity (Zuffa et al. 2024). To evaluate differences in stomatal kinetics and photosynthetic light parameters across SD groups and locations, the TukeyHSD test (‘tukey_hsd’ function from the rstatix package, v.0.7.2) was applied. In these analyses, each parameter was treated as a response variable, with SD group and location as factors.

Relationships among the studied plant traits and climate variables were explored using Linear Mixed Models fitted using the restricted maximum likelihood as implemented in the ‘lmer’ function of the lme4 R package (v.1.1‐36) (Bates et al. 2015). Plant traits (SD, *g*_*s*_, *A*_*net*_, iWUE, δ^13^C, TFW) were tested as a response variable against the other five plant traits and specific environmental conditions as predictor variables [average maximum daily temperature (T_max_), average maximum daily vapor pressure deficit (VPD_max_), and PDSI]. As iWUE is calculated as the ratio between *A*_net_ and g_s_, models combining these variables were excluded to prevent collinearity. Average *T*_max_ and VPD_max_ were calculated by extracting the daily maximum values between 12:00 and 14:00 during the summer months in June, July, August (*T*_max_ Summer, VPD_max_ Summer) and during the spring months in April/May (*T*_max_ Spring, VPD_max_ Spring) at each location. Furthermore, random effects for the genotype and location were tested to account for differences between genotypes and the management or site‐specifics of each location. The significance of fixed effects was assessed through Chi‐square tests using the ‘drop1’ function from lme4 (v.1.1–36), while random effects were evaluated using the ‘ranova’ function from the lmerTest package (v.3.1–3) (Kuznetsova et al. 2017). The *p*‐values for both fixed and random effects were log‐transformed (−log_10_(*p*)) for visualization. The Bonferroni‐corrected significance was calculated as α = α/m with α = 0.05 and m = (6 × 10), which indicates the product of the number of plant traits (equal to 6) by the number of predictors tested for each trait (equal to 10). Therefore, the Bonferroni corrected significance was equal to −log_10_(0.05/(6 × 10)) = 3.08.

## 3 Results

### 3.1 Climatic Trends Across Different Orchard Locations and Growing Seasons

The 2022 growing season exhibited stressful climatic conditions as it was characterized by a comparatively hot and dry climate across the different apple REFPOP European locations in Spain (ESP), France (FRA), Italy (ITA), and Switzerland (CHE). In contrast, 2023 was characterized by cooler temperatures (on average, 4.5°C lower in ESP and 3.5°C lower in FRA) and wetter conditions. Table 1 summarizes key differences in mean maximum daily temperatures (*T*_max_), mean maximum daily VPD (VPD_max_), precipitation and irrigation during sampling weeks between years (2022 and 2023) and locations (ESP, FRA, ITA, CHE). The only locations with repeated sampling in 2023 were ESP and FRA. Longer‐term climate differences across locations, to which the trees have acclimated over many years, are further demonstrated by environmental data from 2019 onward (Figure S1).

**TABLE 1:** Seasonal conditions at the apple REFPOP orchard in Spain (ESP), France (FRA), Italy (ITA), Switzerland (CHE) in 2022 and 2023. The average maximum daily temperature (T_max_, °C) and the average maximum daily vapor pressure deficit (VPD_max_, kPa) were calculated by extracting the daily maximum value between 12:00 and 14:00 during the two (ESP, FRA, ITA) or three (CHE) sampling weeks at each location. Cumulative precipitation (mm) and supplied irrigation (mm) are indicated for the week prior to and during the sampling weeks. ESP, FRA, and ITA applied irrigation; CHE did not. Average monthly potential evapotranspiration (PE_T_, mm/summer month) is reported for the summer season (June, July, and August) at each location. Palmer Drought Severity Index (PDSI, unitless) is reported for the entire summer season (June, July, and August).

Year	Location	T_max_ (°C)	VPD_max_ (kPa)	Precipitation (mm)	Irrigation (mm)	PE_T_ (mm/month)	PDSI
2022	ESP	34.16	4.02	9.1	70.79	194.34	−1.07
2023	ESP	29.66	2.56	41.3	65.60	182.55	−0.80
2022	FRA	27.54	2.36	6.8	34.60	145.88	−0.53
2023	FRA	24.01	1.34	15.2	12.30	128.61	0.84
2022	ITA	30.42	2.09	40.8	35.20	163.77	−0.51
2022	CHE	23.05	1.28	260.8	No irrigation	147.84	0.44

### 3.2 Stomatal Density Remains Consistent Across Different Locations

**FIGURE 1:**
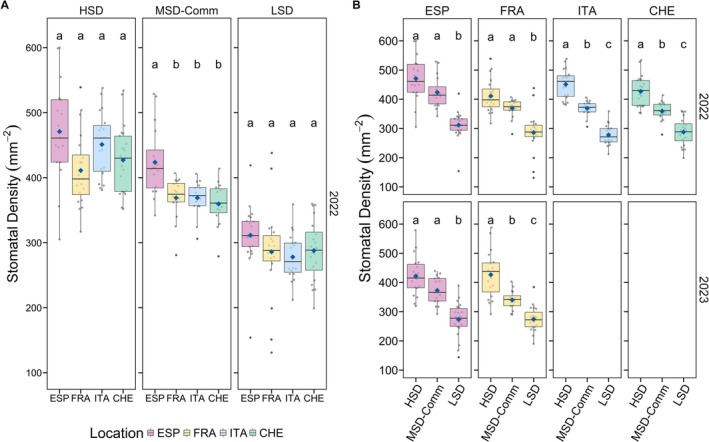
Stomatal density (SD) across locations. Boxplots with means (blue diamonds) and individual tree data points of SD for the selected subsets of accessions in three SD groups (*n* = 258): High SD (HSD), commercial cultivars with medium SD (MSD‐Comm) and low SD (LSD) group. (A) Year 2022 within each SD group across different locations in Spain (ESP, pink colored), France (FRA, yellow colored), Italy (ITA, blue colored), and Switzerland (CHE, green colored) and (B) in 2022 and 2023 between SD groups within each location. Significant differences were assessed using Wilcoxon tests and indicated with different letters (*p* < 0.05, *p*‐values were adjusted with the Bonferroni correction). The box extends from the 1st quartile (25th percentile, lower edge) to the 3rd quartile (75th percentile, upper edge), with the median displayed as a line inside. Whiskers reach the smallest and largest values within 1.5 times the interquartile range, while outliers are shown as individual points beyond the whiskers.

For each of the three SD groups, SD remained stable across locations (*p* > 0.05), with only one exception found in 2022 for the MSD‐Comm group in ESP, which exhibited significantly higher SD values (*p* < 0.05) compared to FRA, ITA and CHE (Figure 1A). Significant differences (*p* < 0.001) between all the three SD groups were found in ITA and CHE in 2022, as well as in FRA in 2023 (Figure 1B). Conversely, no significant differences (*p* > 0.05) were observed between HSD and MSD‐Comm groups in FRA and ESP in 2022 and in ESP in 2023, although they were both significantly different from the LSD group (*p* < 0.05) (Figure 1B). In ESP and FRA, HSD did not show significant differences across locations and years (*p* > 0.05), while MSD‐Comm and LSD have statistical differences between ESP and FRA in each specific location–year combination (Figure S2, *p* < 0.05; 2022 ESP MSD‐Comm is significantly greater than 2022 FRA MSD‐Comm and 2023 FRA MSD‐Comm; 2022 ESP LSD is significantly greater than 2023 FRA LSD). Finally, to assess SD variation across all locations, SD variation among accessions between locations was analysed using a Pearson correlation coefficient (*r*_*p*_). The *r*_*p*_ showed an average value of 0.93 across locations (*p* < 0.001), confirming the high stability of SD in different environments.

### 3.3 Leaf Gas‐Exchange Indicates Physiological Differences Across Locations and Between SD Groups

**FIGURE 2:**
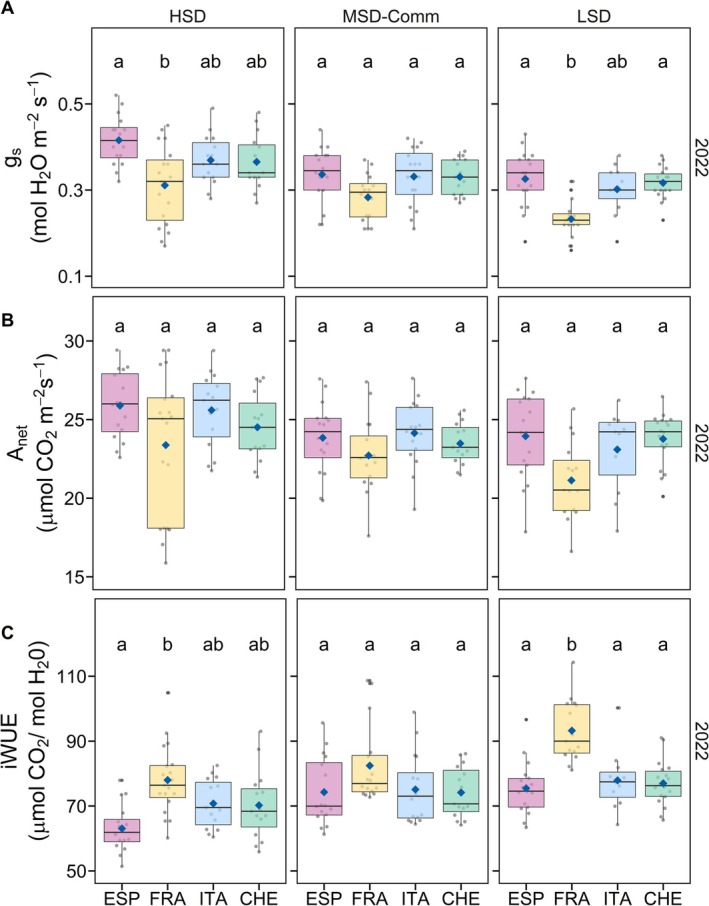
Leaf gas‐exchange measurements indicate key physiological differences across locations within each stomatal density (SD) group. Boxplots with means (blue diamonds) and individual tree data points of (A) stomatal conductance (*g*_*s*_), (B) net carbon assimilation (*A*_*net*_), (C) intrinsic water‐use efficiency (iWUE) (*n* = 189). The box extends from the 1st quartile (25th percentile, lower edge) to the 3rd quartile (75th percentile, upper edge), with the median displayed as a line inside. Whiskers reach the smallest and largest values within 1.5 times the interquartile range, while outliers are shown as individual points beyond the whiskers. Measurements from trees that were lacking fruit were omitted. Measurement conditions were set as follows: Flow rate 600 μmol s^−1^; fan speed 10,000 rpm; [CO_2_] 415 ppm; PAR 1500 μmol photons m^−2^ s^−1^. Measurements were taken between 08:30 and 13:30, depending on the daily weather conditions. Significant differences across locations within each SD group were assessed using Wilcoxon tests and indicated with different letters (*p* < 0.05, *p*‐values were adjusted with the Bonferroni correction).

In 2022, *g*_*s*_ was significantly lower for HSD in FRA compared to ESP and for LSD in FRA compared to ESP and CHE (Figure 2A; *p* < 0.05 for HSD, *p* < 0.001 for LSD). This difference between FRA and other locations was not observed for MSD‐Comm (*p* > 0.05). Across SD groups, *g*_*s*_ showed no significant differences in FRA, ITA, or CHE (Figure S3A, *p* > 0.05); however, in ESP the *g*_*s*_ values for HSD were significantly higher than LSD (Figure S3A; *p* < 0.05) in 2022. By contrast, in 2023, which was characterized by cooler and wetter conditions than 2022 (Table 1, Figure S1), a greater variation in *g*_*s*_ was observed between SD groups in both ESP and FRA (Figure S3A). In these locations, the *g*_*s*_ of HSD was significantly higher (*p* < 0.001) than MSD‐Comm and LSD (Figure S3B). For all SD groups, *g*_*s*_ values were higher in 2023 than in 2022 (Figure S3B, *p* < 0.05). In comparison to the yearly and location‐specific variability in *g*_*s*_, no significant differences in *A*_*net*_ were detected across locations (Figure 2B; *p* > 0.05) or across SD groups within the same location in 2022 and 2023 (Figure S3C; *p* > 0.05). However, *A*_*net*_ was higher overall in 2023 than in 2022 (Figure S3D). Variation in iWUE was primarily influenced by changes in *g*_*s*_ rather than *A*_*net*_ (Figure 2C). Overall, 2022 had higher iWUE compared to 2023 (Figure S3F). For iWUE, no significant differences were found across locations within the MSD‐Comm group, but FRA exhibited significantly higher iWUE compared to ESP within the HSD group and compared to all locations for the LSD group in 2022 (Figure 2C, *p* < 0.001). Similarly, within‐location comparisons revealed no significant differences between SD groups in ITA and CHE (Figure S3E). In FRA, iWUE was significantly higher (*p* < 0.05) for LSD than the other SD groups, while in ESP, the iWUE was lower (*p* < 0.01) for HSD (Figure S3E). When comparing iWUE across years (2022 and 2023) and across locations (ESP and FRA) within each SD group, MSD‐Comm showed significant differences (*p* < 0.001) between years but not across locations (Figure S3F). Conversely, in HSD and LSD groups, significant differences (*p* < 0.001) were observed across locations only in 2022 (Figure S3F).

### 3.4 Stomatal Kinetics Within Each SD Group Are Insensitive to Local Climate

**FIGURE 3:**
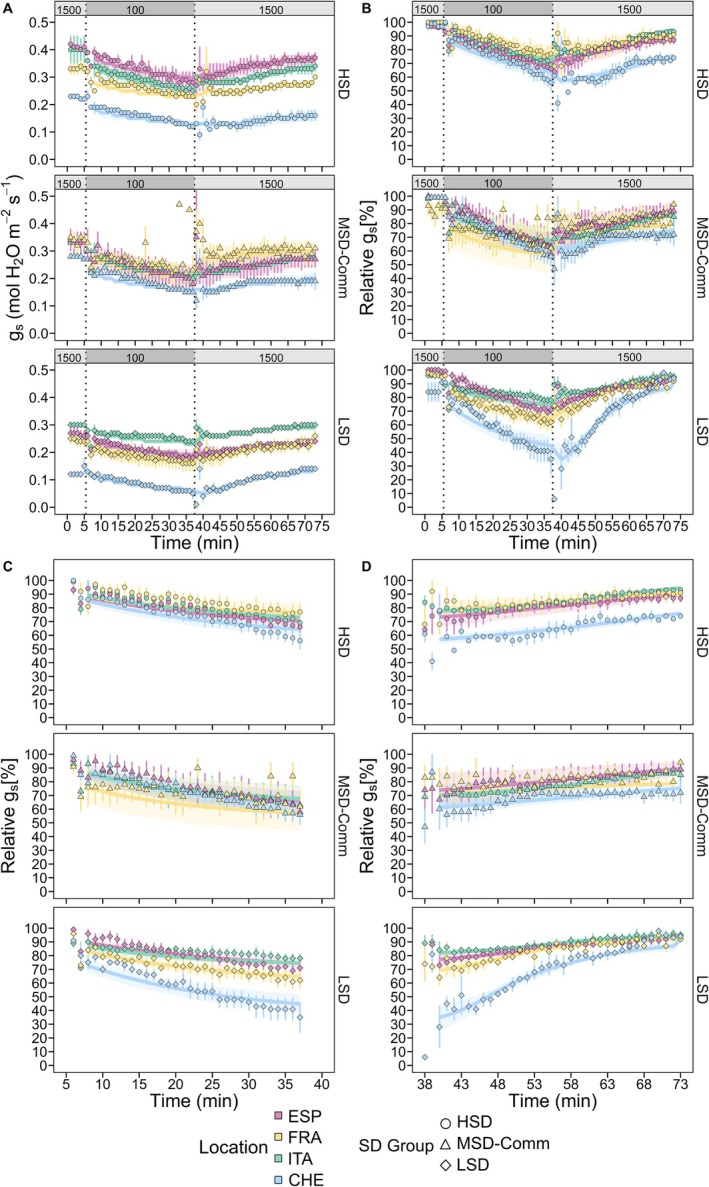
Stomatal kinetics within stomatal density (SD) groups across locations. (A) Absolute response to changes in light intensity of stomatal conductance (*g*_*s*_). (B) Relative response to changes in light intensity of stomatal conductance (Relative *g*_*s*_). (C) Response phase for the light transition from 1500 to 100 PAR of Relative *g*_*s*_. (D) Recovery phase for the light transition from 100 to 1500 PAR for Relative *g*_*s*_. Individual curves in B, C, and D were normalized to the maximum value observed in each replicate. Points represent the mean value of three replications for each accession (*n* = 9) in each SD group: High SD (HSD) (circle chape), commercial cultivars with medium SD (MSD‐Comm) (tringle shape) and low SD (LSD) (diamond shape) in the locations in Spain (ESP, pink colored), France (FRA, yellow colored), Italy (ITA, blue colored), and Switzerland (CHE, green colored). Vertical error bars represent standard error of the mean. Solid lines indicate the mean value of the temporal response model used to predict *g*_*s*_ for each accession (*n* = 9) in each SD group and location. Line confidence intervals represent standard error of the predicted mean.

Absolute ***g***_***s***_ values exhibited differences between locations for HSD and LSD, whereas MSD‐Comm showed minimal ***g***_***s***_ variation (Figures 3A and S4A). In all SD comparisons, ITA consistently had the lowest starting ***g***_***s***_ values under the initial steady‐state conditions and throughout the response curve (Figure 3A). However, once ***g***_***s***_ values were normalized on a relative scale, the differences between locations largely disappeared (Figure 3B–D and S4B).

To test whether the speed of stomatal response differed among SD groups or locations, the time constant ($\tau_{d}$) was calculated, which represents the time required to reach 63% of the total ***g***_***s***_ decline during the response phase (1500 to 100 PAR). Results showed that $\tau_{d}$ did not significantly differ between SD groups ($\tau_{d}$ absolute ***g***_***s***_: *p* = 0.82; $\tau_{d}$ Relative ***g***_***s***_: *p* = 0.94, Figure 3C, Table S2) nor locations ($\tau_{d}$ absolute ***g***_***s***_: *p* = 0.70; $\tau_{d}$ Relative ***g***_***s***_: *p* = 0.94, Figure S4, Table S2). During the recovery phase following the second light transition (100 to 1500 PAR), the speed of stomatal opening, represented by ${\mathrm{Sl}}_{\max}$, showed no significant differences between SD groups (${\mathrm{Sl}}_{\max}$ absolute ***g***_***s***_: *p* = 0.33; ${\mathrm{Sl}}_{\max}$ Relative ***g***_***s***_: *p* = 0.31) nor locations (${\mathrm{Sl}}_{\max}$ absolute ***g***_***s***_: *p* = 0.80; ${\mathrm{Sl}}_{\max}$ Relative ***g***_***s***_: *p* = 0.55, Table S3). Similarly, the time constant ($\tau_{i}$) in this phase was not significantly different across groups ($\tau_{i}$ absolute ***g***_***s***_: *p* = 0.30; $\tau_{i}$ Relative ***g***_***s***_: *p* = 0.21) or locations ($\tau_{i}$ absolute ***g***_***s***_: *p* = 0.89; $\tau_{i}$ Relative ***g***_***s***_: *p* = 0.90, Table S3). These findings suggest that ***g***_***s***_ kinetics (response and recovery phases) are consistent across SD groups and locations, indicating that stomatal dynamics are not influenced by local environment.

To further explore these results, the speed of *A*_*net*_ response was also analyzed by examining the time constant ($\tau_{a}$), which measures the time required to reach 95% of the total change in *A*_*net*_. This analysis revealed that there were no significant differences in the response time between SD groups ($\tau_{a}$ absolute ***A***_***net***_: *p* = 0.47; $\tau_{a}$ Relative ***A***_***net***_: *p* = 0.51, Figure S5, Table S4) nor between locations ($\tau_{a}$ absolute ***A***_***net***_: *p* = 0.45; $\tau_{a}$ Relative ***A***_***net***_: *p* = 0.43, Figure S6, Table S4). Similarly, during the recovery phase, no significant differences were found between SD groups ($\tau_{a}$ absolute ***A***_***net***_: *p* = 0.43; $\tau_{a}$ Relative ***A***_***net***_: *p* = 0.43, Figure S5, Table S5) nor between locations ($\tau_{a}$ absolute ***A***_***net***_: *p* = 0.43; $\tau_{a}$ Relative ***A***_***net***_: *p* = 0.53, Figure S6, Table S5).

### 3.5 Integrated Water‐Use Efficiency, Leaf Nitrogen Content, Tree Growth, and Fruit Yield

**FIGURE 4:**
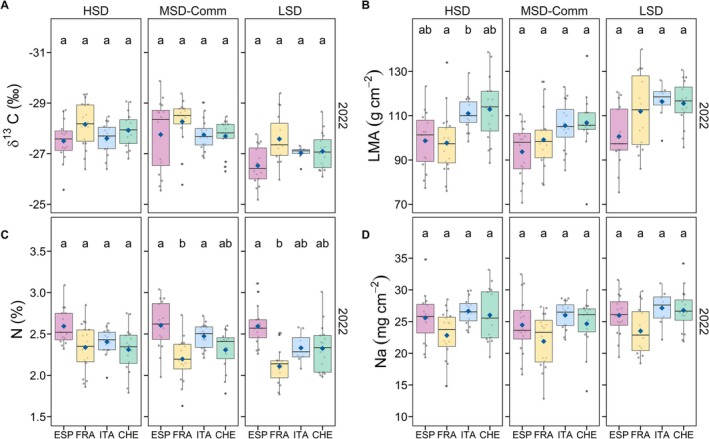
Leaf traits across locations. Boxplots with means (blue diamonds) and individual tree data points of (A) integrated water‐use efficiency (δ^13^C), (B) leaf mass area (LMA), (C) nitrogen percentage (%), (D) nitrogen content per unit leaf area (Na) for each SD group (*n* = 189): High SD (HSD), commercial cultivars with medium SD (MSD‐Comm), and low SD (LSD) in the locations in Spain (ESP, pink colored), France (FRA, yellow colored), Italy (ITA, blue colored), and Switzerland (CHE, green colored). The box extends from the 1st quartile (25th percentile, lower edge) to the 3rd quartile (75th percentile, upper edge), with the median displayed as a line inside. Whiskers reach the smallest and largest values within 1.5 times the interquartile range, while outliers are shown as individual points beyond the whiskers. Measurements from trees that were lacking fruit are omitted. Significant differences across locations within each SD group were assessed using Wilcoxon tests and indicated with different letters (*p* < 0.05, *p*‐values were adjusted with the Bonferroni correction).

δ^13^C showed no significant differences across locations within SD groups (*p* = 0.83, Figure 4A). However, within the same location, a comparison across SD groups revealed that δ^13^C was significantly higher in HSD compared to LSD in both ESP and CHE (*p* < 0.05, Figure S7). Similarly, leaf mass per area (LMA) did not differ significantly across locations within SD groups (*p* = 0.58), except for the HSD group, where significant differences between the locations were observed (*p* < 0.05, Figure 4B). Leaf nitrogen content (N%) was largely consistent across locations within SD groups, with two exceptions: FRA was significantly lower than ITA (*p* < 0.05) and ESP (*p* < 0.05) in MSD‐Comm; and FRA was significantly lower than ESP in LSD (*p* < 0.001, Figure 4C). Nitrogen content per unit leaf area (N_a_) showed no significant differences across locations (*p =* 0.86, Figure 4D).

**FIGURE 5:**
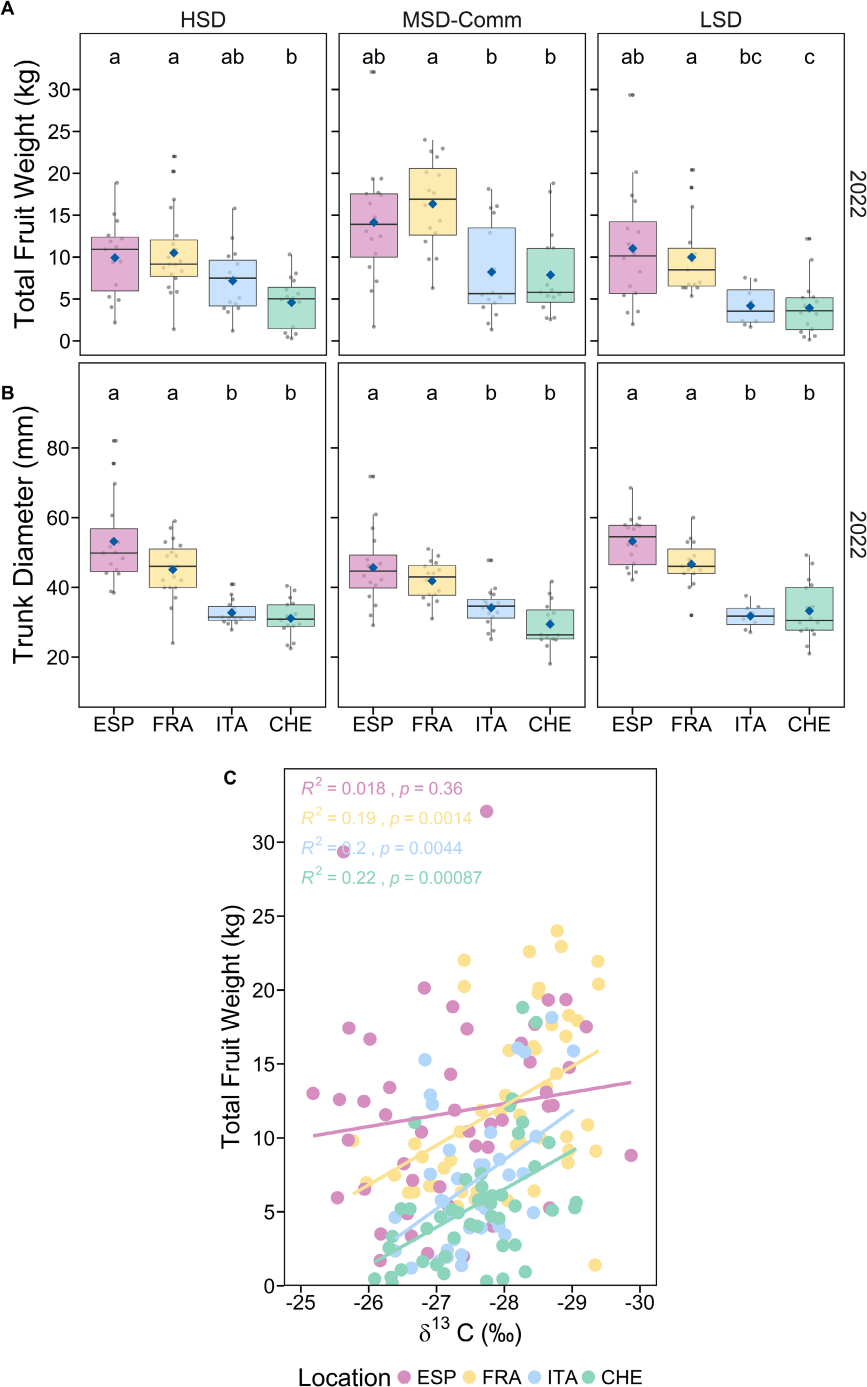
Fruit yields and tree growth across locations. Boxplots with means (blue diamonds) and individual tree data points of (A) Total Fruit Weight, (B) Trunk Diameter in the locations in Spain (ESP, pink colored), France (FRA, yellow colored), Italy (ITA, blue colored), and Switzerland (CHE, green colored) (*n* = 189). The box extends from the 1st quartile (25th percentile, lower edge) to the 3rd quartile (75th percentile, upper edge), with the median displayed as a line inside. Whiskers reach the smallest and largest values within 1.5 times the interquartile range, while outliers are shown as individual points beyond the whiskers. Measurements from trees that were lacking fruit are omitted. Significant differences across locations within each SD group were assessed using Wilcoxon tests and indicated with different letters (*p* < 0.05, *p*‐values were adjusted with the Bonferroni correction). (C) Relationship between TFW and integrated water‐use efficiency (δ^13^C). Regression analysis between TFW and δ^13^C. Each point represents the average accession value at individual locations in ESP (pink colored), FRA (yellow colored), ITA (blue colored), and CHE (green colored) in 2022.

TFW and TD were selected to represent yield and vigor, respectively, and these traits showed differences between SD groups across locations (Figure 5). TFW revealed considerable location‐based differences (Figures 5A and S8): FRA and ESP had significantly greater TFW for both HSD and LSD in 2022 than CHE (Figure 5A; *p < 0.05*) with the exception being MSD‐Comm. FRA had significantly greater TFW than ITA within LSD (*p* < 0.05) and MSD‐Comm (*p* < 0.01). Within each location, TFW showed no significant differences between SD groups (*p* = 0.96), except in FRA, where MSD‐Comm was significantly greater than both HSD and LSD (*p* < 0.05, Figures 5A and S9). Significant location‐based variation was observed for TD (Figure 5B): all SD groups in FRA and ESP had significantly larger TD compared to CHE and ITA (*p* < 0.001, Figure 5B). However, no differences in TD were observed among SD groups within each location (*p* = 0.91, Figure S9). Additionally, TFW had a positive correlation with δ^13^C in FRA, ITA and CHE (*p* < 0.001), while no correlation was observed in ESP (*p* = 0.36, Figure 5C).

### 3.6 Relationships Between Growth Environment, Stomatal Traits, δ^13^C and Total Fruit Weight

Linear mixed model analyses explored the potential relationships between plant traits (SD, *g*_*s*_, *A*_*net*_, iWUE, δ^13^C, TFW), local climate variables (T_max_ Spring, T_max_ Summer, VPD_max_ Spring, VPD_max_ Summer, PDSI), genotype and orchard location (Table 2). In all response and predictor variable combinations, the random effects of genotype were significant. When SD was the response variable, *A*_*net*_ was the only significant fixed effect (−log_10_(*p*‐value) = 3.61*). The random effects of location were significant with SD as the response variable and PDSI, *g*_*s*_, *A*_*net*_, iWUE, δ^13^C, TFW as the predictor variables (−log_10_(*p*‐value) = 6.47*, 3.15*, 3.73*, 4.69*, 5.79* and 5.39*, respectively). When *g*_*s*_ was the response variable, *A*_*net*_ and δ^13^C were significant fixed effects (−log_10_(*p*‐value) = 52.93* and 3.81* respectively). The random effects of location were always significant with *g*_*s*_ as the response variable. When δ^13^C was the response variable, *g*_*s*_, iWUE and TFW were significant fixed effects (−log_10_(*p*‐value) = 3.41*, 4.39*, and 3.31* respectively). The random effects of location were significant with δ^13^C as the response variable and *g*_*s*_, *A*_*net*_, iWUE, δ^13^C, TFW as the predictor variables (−log_10_(*p*‐value) = 4.70*, 7.32*, 5.62*, 8.27*, and 4.26*, respectively). Finally, when TFW was the response variable, only δ^13^C had significant fixed effects (−log_10_(*p*‐value) = 3.45*). The random effects of location were always significant with TFW as the response variable (−log_10_(*p*‐value) > 5.03*).

**TABLE 2:** The –log_10_ transformed *p*‐values for fixed and random effects in linear mixed models to assess the significance of the relationship between plant traits, environmental conditions, genotype and location are shown. Measured plant traits include stomatal density (SD), stomatal conductance (*g*_*s*_), net carbon assimilation (*A*_*net*_), intrinsic water‐use efficiency (iWUE), integrated water‐use efficiency (δ^13^C), and total fruit weight (TFW). Environmental conditions include average maximum daily temperature (T_max_) in spring (April, May) and summer (June, July, August), average maximum daily vapor pressure deficit (VPD_max_ spring and VPD_max_ summer), and the Palmer Drought Severity Index (PDSI). All plant traits are treated as a response variable and predictor fixed effect, while environmental conditions are only considered as predictor fixed effects. Genotype and location are only treated as random effects throughout. *p*‐values for fixed effects were calculated using a ‘Chi‐square’ test and *p*‐values for random effects were calculated using likelihood ratio tests. The Bonferroni‐corrected significance was calculated as *α* = α/m with *α* = 0.05 and m = (6 10), which represents the product of the number of plant traits (equal to 6) by the number of predictors tested (equal to 10). Therefore, the Bonferroni corrected significance was equal to −log_10_(0.05/(6 × 10)) = 3.08. Significant −log_10_(*p*) are indicated with (*).

Response	Predictor	Fixed effects (−log_10_(*p*))	Random effects Genotype (−log_10_(*p*))	Random effects Location (−log_10_(*p*))
SD	T_max_ Spring	1.43	31.64*	1.28
SD	T_max_ Summer	1.64	31.72*	0.87
SD	VPD_max_ Spring	2.76	32.46*	0.00
SD	VPD_max_ Summer	2.89	32.59*	0.00
SD	PDSI	0.12	32.00*	6.47*
SD	*g*_*s*_	1.97	28.22*	3.15*
SD	*A*_*net*_	3.61*	31.66*	3.73*
SD	iWUE	0.13	28.19*	4.69*
SD	δ^13^C	0.02	30.00*	5.79*
SD	TFW	0.98	31.75*	5.39*
*g*_*s*_	T_max_ Spring	0.85	6.78*	6.55*
*g*_*s*_	T_max_ Summer	1.16	6.78*	4.75*
*g*_*s*_	VPD_max_ Spring	1.47	6.79*	3.44*
*g*_*s*_	VPD_max_ Summer	1.31	6.78*	4.18*
*g*_*s*_	PDSI	0.93	6.75*	5.91*
*g*_*s*_	SD	2.77	4.33*	8.47*
*g*_*s*_	*A*_*net*_	52.93*	11.06*	7.86*
*g*_*s*_	δ^13^C	3.81*	5.15*	13.78*
*g*_*s*_	TFW	1.27	7.44*	11.61*
*A*_*net*_	T_max_ Spring	1.00	3.74*	1.89
*A*_*net*_	T_max_ Summer	1.34	3.76*	1.17
*A*_*net*_	VPD_max_ Spring	1.68	3.73*	0.70
*A*_*net*_	VPD_max_ Summer	1.50	3.72*	0.97
*A*_*net*_	PDSI	0.78	3.71*	2.41
*A*_*net*_	SD	3.33*	3.68*	2.58
*A*_*net*_	*g*_*s*_	53.03*	7.49*	1.00
*A*_*net*_	δ^13^C	1.51	4.13*	5.02*
*A*_*net*_	TFW	1.28	4.55*	4.55*
iWUE	Tmax Spring	0.64	11.43*	10.23*
iWUE	Tmax Summer	0.89	11.43*	8.12*
iWUE	VPDmax Spring	1.08	11.45*	6.88*
iWUE	VPDmax Summer	0.95	11.45*	7.87*
iWUE	PDSI	1.29	11.39*	5.28*
iWUE	SD	0.68	8.43*	11.99*
iWUE	δ^13^C	4.71*	7.59*	17.66*
iWUE	TFW	1.00	11.57*	14.51*
δ^13^C	T_max_ Spring	1.00	11.56*	2.12
δ^13^C	T_max_ Summer	1.26	11.54*	1.44
δ^13^C	VPD_max_ Spring	1.03	11.44*	1.91
δ^13^C	VPD_max_ Summer	0.87	11.45*	2.35
δ^13^C	PDSI	1.01	11.57*	1.99
δ^13^C	SD	0.50	9.91*	4.70*
δ^13^C	*g*_*s*_	3.41*	9.92*	7.32*
δ^13^C	*A*_*net*_	1.63	12.01*	5.62*
δ^13^C	iWUE	4.39*	7.91*	8.27*
δ^13^C	TFW	3.31*	9.89*	4.26*
TFW	T_max_ Spring	0.33	6.30*	10.90*
TFW	T_max_ Summer	0.24	6.30*	11.56*
TFW	VPD_max_ Spring	0.36	6.30*	10.96*
TFW	VPD_max_ Summer	0.46	6.30*	10.29*
TFW	PDSI	1.22	6.31*	5.03*
TFW	SD	0.87	6.39*	11.16*
TFW	*g*_*s*_	0.99	6.77*	12.23*
TFW	*A*_*net*_	1.33	7.14*	12.24*
TFW	iWUE	0.81	6.36*	12.17*
TFW	δ^13^C	3.45*	4.71*	11.50*

## 4 Discussion

### 4.1 Stomatal Density Remains Consistent Across Locations but Can Acclimate to Stressful Environments

Our study revealed that SD exhibited stability across different locations, with only a few exceptions. The MSD‐Comm group showed significantly higher SD in 2022 in ESP than in other locations (Figure 1A). Elevated temperatures coupled with high irrigation rates in ESP (Table 1, Figure S1) resulted in the highest mean SD value in each SD group (Figure 1A). This pattern suggests that increased SD has greater potential for evaporative leaf cooling, potentially counteracting the damaging effects of photorespiration and fulfilling the need for leaf heat dissipation (Berry and Bjorkman 1980; Doheny‐Adams et al. 2012). These findings align with previous research suggesting that moderate heat stress can trigger an increase in SD in rice ( *Oryza sativa* Wu et al. 2020), basil ( *Ocimum basilicum* Driesen et al. 2023), *Brachypodium distachyon* L. (Nunes et al. 2022) and poplar (*Populus* spp., Pearce et al. 2006). In contrast, FRA had a significant reduction in the HSD group in 2022 compared to 2023, despite experiencing comparable heat stress in 2022 (Figures 1A and S1; Table 1). This reduction might indicate a more conservative developmental response in trees that experience heat exposure with a lower amount of water provided through irrigation. However, this reduction in HSD reversed itself in 2023, whereby HSD was significantly different from MSD‐Comm in FRA and the mean HSD values between ESP and FRA were nearly identical (Figure 1B and S2). This demonstrates the capacity of SD to react on a yearly basis to stress conditions without inducing permanent changes. This aligns with prior research that SD plasticity can be an important mechanism for plants to cope with increasing heat and diminishing water availability (Bertolino et al. 2019; Gray and Dunn 2024). In contrast, acclimation of HSD and MSD‐Comm in ESP appears more stable: HSD and MSD‐Comm values were not significantly different in either 2022 or 2023 (Figure 1B). The relatively mild conditions in ITA and CHE had distinct SD group differences (HSD, MSD‐Comm, and LSD) in 2022 (Figure 1B) that reflect the previously observed trend in CHE, where significant SD group differences were reported between 2019 and 2022 (Zuffa et al. 2024). While our findings do not conclusively establish causation between heat‐induced developmental acclimation and higher SD, they do demonstrate that SD in trees is relatively stable across environments and can adjust to seasonal environmental stress under extreme conditions. This contradicts our initial hypothesis that SD would be highly responsive to local conditions, which was based on various studies in controlled environments. This stability across locations, combined with the previously reported low interannual variability (Zuffa et al. 2024), confirms the value of SD as a breeding trait to screen for improved abiotic stress resilience. Although our study focused on accessions with extreme SD phenotypes, intermediate genotypes were also included (20% of the analyzed genotypes were MSD‐Comm). Moreover, the response observed in HSD genotypes in FRA 2022 suggests that extreme genotypes are not developmentally fixed, but can still respond to local environmental stress. Together, these observations indicate that our dataset captures a range of genotypic responses, while still acknowledging that results can be interpreted in the context of the selected subset of genotypes.

### 4.2 Stomatal Physiology Impacts Environmental Acclimation

On average, ESP exhibited lower iWUE and higher *g*_*s*_ compared to other locations, particularly FRA, which showed the highest iWUE and lowest *g*_*s*_ (Figure 2A,C). This indicates more efficient water utilization via reduced gas exchange rates in FRA. The longer‐term physiological difference between FRA and other locations might be exaggerated due to the sampling time period in FRA 2022, which coincided with the hottest and driest part of the summer and likely contributed to the large reductions in *g*_*s*_ (T_max_ reached average highs of 38.54°C in the late afternoon, although T_max_ only reached 27.54°C during gas‐exchange measurements; Table 1). High temperatures were also present during the sampling time period in ESP 2022 (late afternoon T_max_ = 40.60°C, sampling T_max_ = 34.16°C; Table 1), but these climatic conditions are more typical for that region and may not induce as dramatic physiological plant responses. In 2023, which was a cooler and wetter year, both FRA and ESP experienced an increase in *g*_*s*_ across all SD groups (Figure S3D), coupled with a decrease in iWUE (Figure S3C). These results suggest that despite the anatomical differences among SD groups, they exhibit similar adaptive capacities to local environmental conditions. This aligns with previous findings that emphasize the role of stomatal function in systemic acclimation to local abiotic stress (Bertolino et al. 2019).

Seasonal differences across locations further highlight the functional plasticity of *g*_*s*_ relative to *A*_*net*_ and emphasize the critical role of stomata in plant environmental acclimation. Stability in *A*_*net*_ across SD groups and locations during 2022 (Figure 2B) suggests that apple trees maintain a baseline level of carbon assimilation. Consequently, variations in iWUE across locations and SD groups in 2022 appear to reflect environmental acclimation of *g*_*s*_ to local climatic and management conditions (Figure 2A,C). Notably, the non‐significant random effect of location in the interactions between *A*_*net*_ with other stomatal traits (*g*_*s*_, iWUE) (Table 2) is consistent with the absence of significant differences in *A*_*net*_ across SD groups in 2022 (Figure 3B). These findings are consistent with previous results suggesting that the combined effects of *g*_*s*_ and *A*_*net*_ on δ^13^C could be used to predict fruit production (Zuffa et al. 2024). Although no statistical differences in δ^13^C were detected across locations in 2022 at the SD group level (Figure 4A), significant relationships were observed when larger sample sizes without SD group distinction were considered (Figure 5, Table 2). Local conditions in FRA, ITA, and CHE led to greater stomatal closure and reduced *g*_*s*_ (Figure 2A), potentially contributing to the observed significant correlation between δ^13^C and TFW within these locations (Figure 5C). Furthermore, the only predictor variable that had significant fixed effects on TFW was δ^13^C (Table 2). In contrast, well‐irrigated conditions in ESP allowed for higher *g*_*s*_ (Figure 2A; Table 1) and maintained high TFW without a significant correlation to δ^13^C (Figure 5C). Higher TFW, that is, fruit crop load, can act as a stronger carbon sink and therefore increase the demand for photosynthesis, requiring greater CO_2_ uptake and potentially more open stomata. Increased stomatal opening can raise intercellular CO_2_ concentration (Ci), which results in more negative δ^13^C values and reduced iWUE. Although the correlation between TFW and δ^13^C alone does not confirm this mechanism, it does provide a plausible explanation for the observed physiology. These findings collectively highlight the critical role of stomatal regulation and WUE in plant acclimation to diverse environmental conditions, emphasizing the interplay between stomatal development, water availability, and carbon assimilation as key determinants of crop productivity.

### 4.3 Environmental Acclimation Is Not Influenced by Stomatal Kinetics

Despite the anatomical differences across SD groups and functional acclimation to local environmental conditions, stomatal response kinetics were consistent and not significantly different across SD groups (Figures S4 and S6) nor across locations (Figures 3 and S5). All SD groups across locations exhibited similar relative rates of stomatal opening and closure when exposed to different light conditions (Tables S2 and S3). This suggests that, while SD defines the absolute range of stomatal conductance, it does not significantly affect the relative speed or efficiency of stomatal responses to light fluctuation. The insensitivity of stomatal kinetics to SD supports prior research highlighting that other stomatal features influence response speediness independently of size and number, such as the number and dimensions of auxiliary cells to facilitate solute exchange (Franks and Farquhar 2007), or differences in biochemical composition and gene expression (Lawson and Blatt 2014). This insensitivity appears particularly true for the kidney‐shaped stomata present in apple (Elliott‐Kingston et al. 2016; McAusland et al. 2016).

### 4.4 Roles of Stomatal Density and Function in Plant Breeding and Fruit Production

Given the observed relationships between stomatal development, leaf physiological processes, and environmental variables, our results indicate that SD plays a role in environmental acclimation via *g*_*s*_. Seasonal adjustment of higher SD at hotter locations can increase the operational capacity of *g*_*s*_ and the ability for leaf cooling under elevated temperatures, and may help sustain carbon fixation that might otherwise limit growth and metabolism (Rogiers et al. 2011). Conversely, genotypes with lower SD can conserve water without a carbon assimilation penalty under benign conditions to improve water management or reduce the need for irrigation. While *g*_*s*_ can directly alter short‐term acclimation, the stability of SD emerges as a key trait for long‐term genetic adaptation, with some seasonal adjustments possible due to developmental plasticity. Moreover, the genetic determinants of SD identified by (Zuffa et al. 2026) and functional validation studies in other crops (Hughes et al. 2017; Yin et al. 2017; Caine et al. 2019; Dunn et al. 2019; Bheemanahalli et al. 2021; Clemens et al. 2022; Rathnasamy et al. 2023) highlight the potential of using SD as a target trait for apple breeding.

Despite the compelling roles of SD and *g*_*s*_, fruit production traits like TFW result from the integration of multiple processes under the influence of genotype, environment and management factors. This is reflected in the MSD‐Comm genotypes that were selected from the larger population to compare the preferred modern production varieties with HSD and LSD counterparts. While the stomatal characteristics of MSD‐Comm genotypes were intermediate to HSD and LSD values, other examined functional traits of MSD‐Comm were not predictive of TFW. The MSD‐Comm group produced significantly more fruit than HSD and LSD genotypes at every site and year (Figure S8). In addition to the studied genetic and environmental factors, site‐specific management practices that were confounded with environmental effects in the apple REFPOP experimental design could also strongly influence fruit production. For example, FRA had the lowest values of *g*_*s*_ and *A*_*net*_ of any location, but the highest fruit production rate of the MSD‐Comm genotypes. This disconnection between leaf function and fruit production is likely the result of specialized management practices that are optimized for the growing region. Fine‐tuning the interplay between leaf functional traits (carbon source), fruit development (carbon sink), and management practices (source and sink manipulation) will ultimately determine production outcomes in a changing climate.

The capacity of SD to impact plant acclimation and adaptation makes it an important trait to be considered in future breeding strategies that aim to improve tree crop resilience. Relative stability of SD across locations and years also provides a reliable phenotype that can be predictably measured. SD could even be tailored to the needs of the cultivation area—whether to maximize carbon assimilation and heat mitigation via higher SD or to improve water‐use efficiency with lower SD.

## Author Contributions

Graham Dow conceived the research plan; Francesca Zuffa performed experiments, data collection, and data analysis; Michaela Jung assisted with the data analysis; Carles Quesada‐Traver and Steven Yates created the automated stomatal counting model; Maria José Aranzana, Lidia Lozano, François Laurens, Hélène Muranty, Elias Holzknecht, and Walter Guerra contributed to data collection. Bruno Studer, Andrea Patocchi, Michaela Jung and Graham Dow supervised the project; Francesca Zuffa interpreted the data and wrote the manuscript together with Graham Dow; all authors edited and approved the final manuscript.

## Funding

This work was supported by Horizon 2020 Framework Programme, 847585; Eidgenössische Technische Hochschule Zürich, ETH‐32 21‐1.

## Conflicts of Interest

The authors declare no conflicts of interest.

## Supporting information


**Table S1:** Raw accession trait data, as collected by location (Spain (ESP), France (FRA), Italy (ITA), Switzerland (CHE)) in 2022 and 2023. Separate file.

**Table S2:** Stomatal conductance (*g*_*s*_) kinetics parameters extracted from the exponential (Equation 2) when the light was decreased to 100 PAR for a set period of 30 min (response phase) for each replicate of each studied genotype in each location Spain (ESP), France (FRA), Italy (ITA), Switzerland (CHE).
**Table S3:** Stomatal conductance (*g*_*s*_) kinetics parameters extracted from the sigmoidal equation (Equation 3) when light was increased back again to 1500 PAR for 30 min (recovery phase) for each replicate of each studied genotype in each location Spain (ESP), France (FRA), Italy (ITA), Switzerland (CHE).
**Table S4:** Net carbon assimilation (*A*_*net*_) time constant ($\tau_{a}$) to reach 95% of the steady‐state *A*_*net*_ when light was decreased from 1500 PAR to 100 PAR for a set period of 30 min (response phase) for each replicate of each studied genotype in each location Spain (ESP), France (FRA), Italy (ITA), Switzerland (CHE).
**Table S5:** Net carbon assimilation (*A*_*net*_) time constant ($\tau_{a}$) to reach 95% of the steady‐state *A*_*net*_ when light was increased from 100 PAR to 1500 PAR for a set period of 30 min (recovery phase) for each replicate of each studied genotype in each location Spain (ESP), France (FRA), Italy (ITA), Switzerland (CHE).
**Figure S1:** Growing season conditions in the apple REFPOP orchard in Spain (ESP), France (FRA), Italy (ITA), Switzerland (CHE) from 2019 to 2023. Lines indicate (A) the average maximum daily temperature (right y‐axis) and (B) the average maximum daily vapor pressure deficit (VPD). These variables were calculated by extracting the daily maximum value between 12:00 and 14:00 and averaging across the month. Bars in (A) indicate the cumulative monthly precipitation and (B) the supplied irrigation (left y‐axis). ESP, FRA, and ITA applied irrigation, CHE did not. Irrigation data were available for ESP, FRA, and ITA in 2022, and for ESP and FRA in 2023. Red‐highlighted bars in 2022 and in 2023 indicate the months during which the leaf physiological measurements sampling was conducted at each specific location.
**Figure S2:** Stomatal density (SD) remains consistent across locations and across years. Boxplots with means (blue diamonds) and individual tree data points of SD for the selected subsets of accessions in three SD groups (*n* = 110): high SD (HSD), commercial cultivars with medium SD (MSD‐Comm) and low SD (LSD) in 2022 and in 2023 across two locations: Spain (ESP, pink colored) and France (FRA, yellow colored). Significant differences across years and locations within each SD group were assessed using Wilcoxon tests and indicated with different letters (*p* < 0.05, *p*‐values were adjusted with the Bonferroni correction). The box extends from the 1st quartile (25th percentile, lower edge) to the 3rd quartile (75th percentile, upper edge), with the median displayed as a line inside. Whiskers reach the smallest and largest values within 1.5 times the interquartile range, while outliers are shown as individual points beyond the whiskers.
**Figure S3:** Leaf gas‐exchange measurements across stomatal density (SD) groups, locations and years. Boxplots with means (blue diamonds) and individual tree data points of (A, B) stomatal conductance (*g*_*s*_), (C, D) net carbon assimilation (*A*_*net*_), (E, F) intrinsic water‐use efficiency (iWUE) (*n* = 274). (A, C, E) across the SD groups: high SD (HSD), commercial cultivars with medium SD (MSD‐Comm) and low SD (LSD); in the locations in Spain (ESP, pink colored), France (FRA, yellow colored), Italy (ITA, blue colored), Switzerland (CHE, green colored), in 2022 and 2023; (B, D, F) across years and locations within each SD group. The box extends from the 1st quartile (25th percentile, lower edge) to the 3rd quartile (75th percentile, upper edge), with the median displayed as a line inside. Whiskers reach the smallest and largest values within 1.5 times the interquartile range, while outliers are shown as individual points beyond the whiskers. Measurements from trees that were lacking fruit are omitted. Measurement conditions were set as follows: flow rate 600 μmol s^−1^; fan speed 10,000 rpm; [CO_2_] 415 ppm; PAR 1500 μmol photons m^−2^ s^−1^. Measurements were taken between 08:30 and 13:30, depending on the daily weather conditions. Significant differences across SD group within each location and year (A, C, E) and across years and locations within each SD group (B, D, F) were assessed using Wilcoxon tests and indicated with different letters (*p* < 0.05, *p*‐values were adjusted with the Bonferroni correction).
**Figure S4:** Stomatal kinetics across locations. Absolute response to changes in light intensity of (A) stomatal conductance (*g*_*s*_). Relative response to changes in light intensity of stomatal conductance (Relative *g*_*s*_) (B). Individual curves were normalized to the maximum value observed in each replicate. Response phase for the light transition from 1500 to 100 PAR of (C) Relative *g*_*s*_. Recovery phase for the light transition from 100 to 1500 PAR for (D) Relative *g*_*s*_. Points represent the mean value of three replications for each accession (*n* = 9) in each SD group: high SD (HSD) (represented by circles with dark color shading), commercial cultivars with medium SD (MSD‐Comm) (triangles with medium color shading) and low SD (LSD) (diamonds with light color shading) in the locations in Spain (ESP, pink colored), France (FRA, yellow colored), Italy (ITA, blue colored), Switzerland (CHE, green colored). Vertical error bars represent the standard error of the mean. Solid lines indicate the mean value of the temporal response model used to predict *g*_*s*_ for each accession (*n* = 9) in each SD group and location. Line confidence intervals represent standard error of the predicted mean.
**Figure S5:** Net carbon assimilation (*A*_*net*_) kinetics within stomatal density (SD) groups across locations. Absolute response to changes in light intensity of (A) net carbon assimilation (*A*_*net*_). Relative response to changes in light intensity of (B) net carbon assimilation (Relative *A*_*net*_). Individual curves were normalized to the maximum value observed in each replicate. Response phase for the light transition from 1500 to 100 PAR of (C) Relative *A*_*net*_. Recovery phase for the light transition from 100 to 1500 PAR for (D) Relative *A*_*net*_. Points represent the mean value of three replications for each accession (*n* = 9) in the locations in Spain (ESP, pink colored), France (FRA, yellow colored), Italy (ITA, blue colored), and Switzerland (CHE, green colored). Vertical error bars represent standard error of the mean. Solid lines indicate the mean value of the temporal response model used to predict *g*_*s*_ for each accession (*n* = 9) in each SD group and location. Line confidence intervals represent standard error of the predicted mean.
**Figure S6:** Net carbon assimilation (*A*_*net*_) kinetics across locations. Absolute response to changes in light intensity of (A) net carbon assimilation (*A*_*net*_). Relative response to changes in light intensity of (B) net carbon assimilation (Relative *A*_*net*_). Individual curves were normalized to the maximum value observed in each replicate. Response phase for the light transition from 1500 to 100 PAR of (C) Relative *A*_*net*_. Recovery phase for the light transition from 100 to 1500 PAR for (D) Relative *A*_*net*_. Points represent the mean value of three replications for each accession (*n* = 9) in each SD group: high SD (HSD) (represented by circles with dark color shading), commercial cultivars with medium SD (MSD‐Comm) (triangles with medium color shading) and low SD (LSD) (diamonds with light color shading) in each location in Spain (ESP, pink colored), France (FRA, yellow colored), Italy (ITA, blue colored), and Switzerland (CHE, green colored). Vertical error bars represent standard error of the mean. Solid lines indicate the mean value of the temporal response model used to predict *g*_*s*_ for each accession (*n* = 9) in each SD group and location. Line confidence intervals represent standard error of the predicted mean.
**Figure S7:** Leaf traits across SD groups within each location. Boxplots with means (blue diamonds) and individual tree data points of (A) integrated water‐use efficiency (δ^13^C), (B) leaf mass area (LMA), (C) nitrogen percentage (%), (D) nitrogen content per unit leaf area (Na) within each location in Spain (ESP, pink colored), France (FRA, yellow colored), Italy (ITA, blue colored), and Switzerland (CHE, green colored) between the SD group (*n* = 189): high SD (HSD), commercial cultivars with medium SD (MSD‐Comm), and low SD (LSD). The box extends from the 1st quartile (25th percentile, lower edge) to the 3rd quartile (75th percentile, upper edge), with the median displayed as a line inside. Whiskers reach the smallest and largest values within 1.5 times the interquartile range, while outliers are shown as individual points beyond the whiskers. Measurements from trees that were lacking fruit are omitted. Significant differences across SD group within each location were assessed using Wilcoxon tests and indicated with different letters (*p* < 0.05, *p*‐values were adjusted with the Bonferroni correction).
**Figure S8:** Total Fruit Weight from 2019 to 2023 within stomatal density (SD) groups across locations. Boxplots with means (blue diamonds) and individual tree data points of Total Fruit Weight (TFW) from 2019 to 2023 for each SD group (*n* = 1702): high SD (HSD), commercial cultivars with medium SD (MSD‐Comm) and low SD (LSD) in the locations in Spain (ESP, pink colored), France (FRA, yellow colored), Italy (ITA, blue colored), and Switzerland (CHE, green colored). The box extends from the 1st quartile (25th percentile, lower edge) to the 3rd quartile (75th percentile, upper edge), with the median displayed as a line inside. Whiskers reach the smallest and largest values within 1.5 times the interquartile range, while outliers are shown as individual points beyond the whiskers. Bearing and not bearing trees were included. Significant differences across locations within each SD group were assessed using Wilcoxon tests and indicated with different letters (*p* < 0.05, *p*‐values were adjusted with the Bonferroni correction).
**Figure S9:** Fruit yields and tree growth across SD groups within each location. Boxplots with means (blue diamonds) and individual tree data points of (A) Total Fruit Weigh, (B) Trunk Diameter within each locations in Spain (ESP, pink colored), France (FRA, yellow colored), Italy (ITA, blue colored), and Switzerland (CHE, green colored) between the SD group (*n* = 189): high SD (HSD), commercial cultivars with medium SD (MSD‐Comm), and low SD (LSD). The box extends from the 1st quartile (25th percentile, lower edge) to the 3rd quartile (75th percentile, upper edge), with the median displayed as a line inside. Whiskers reach the smallest and largest values within 1.5 times the interquartile range, while outliers are shown as individual points beyond the whiskers. Measurements from trees that were lacking fruit are omitted. Significant differences across SD group within each location were assessed using Wilcoxon tests and indicated with different letters (*p* < 0.05, *p*‐values were adjusted with the Bonferroni correction).

## Data Availability

All data generated for this study in 2022 and 2023 across different REFPOP locations are available in Table S1, listed by accession and replicate tree.
