# Supplementary material for: Limited Plasticity of Stomatal Development in Apple Trees Across Diverse European Climates
Source: Physiol Plant. 2026 Jul 5;178(4):e71000. doi: 10.1111/ppl.71000 (PMC13333600; doi:10.1111/ppl.71000)
Supplement: Supplementary file 2 — Table S2: Stomatal conductance (g s ) kinetics parameters extracted from the exponential (Equation 2) when the light was decreased to 100 PAR for a set period of 30 min (response phase) for each replicate of each studied genotype in each location Spain (ESP), France (FRA), Italy (ITA), Switzerland (CHE). Table S3: Stomatal conductance (g s ) kinetics parameters extracted from the sigmoidal equation (Equation 3) when light was increased back again to 1500 PAR for 30 min (recovery phase) for each replicate of each studied genotype in each location Spain (ESP), France (FRA), Italy (ITA), Switzerland (CHE). Table S4: Net carbon assimilation (A net ) time constant (τa) to reach 95% of the steady‐state A net when light was decreased from 1500 PAR to 100 PAR for a set period of 30 min (response phase) for each replicate of each studied genotype in each location Spain (ESP), France (FRA), Italy (ITA), Switzerland (CHE). Table S5: Net carbon assimilation (A net ) time constant (τa) to reach 95% of the steady‐state A net when light was increased from 100 PAR to 1500 PAR for a set period of 30 min (recovery phase) for each replicate of each studied genotype in each location Spain (ESP), France (FRA), Italy (ITA), Switzerland (CHE). Figure S1: Growing season conditions in the apple REFPOP orchard in Spain (ESP), France (FRA), Italy (ITA), Switzerland (CHE) from 2019 to 2023. Lines indicate (A) the average maximum daily temperature (right y‐axis) and (B) the average maximum daily vapor pressure deficit (VPD). These variables were calculated by extracting the daily maximum value between 12:00 and 14:00 and averaging across the month. Bars in (A) indicate the cumulative monthly precipitation and (B) the supplied irrigation (left y‐axis). ESP, FRA, and ITA applied irrigation, CHE did not. Irrigation data were available for ESP, FRA, and ITA in 2022, and for ESP and FRA in 2023. Red‐highlighted bars in 2022 and in 2023 indicate the months during which the leaf physiological measurem [file PPL-178-e71000-s002.docx]

**Supplementary Data**

**Table S1:** Raw accession trait data, as collected by location (Spain (ESP), France (FRA), Italy (ITA), Switzerland (CHE)) in 2022 and 2023. Separate file.

**Table S2:** Stomatal conductance (*g_s_*) kinetics parameters extracted from the exponential equation (Eqn 2) when the light was decreased to 100 PAR for a set period of 30 minutes (response phase) for each replicate of each studied genotype in each location Spain (ESP), France (FRA), Italy (ITA), Switzerland (CHE).

| $\boldsymbol{G}_{\boldsymbol{max}}$  (*g*_s_) | $\boldsymbol{G}_{\boldsymbol{min}}$  (*g*_s_) | $\boldsymbol{\tau}_{\boldsymbol{d}}$  (*g*_s_) | $\boldsymbol{G}_{\boldsymbol{max}}$ (Relative *g*_s_) | $\boldsymbol{G}_{\boldsymbol{min}}$ (Relative *g*_s_) | $\boldsymbol{\tau}_{\boldsymbol{d}}$  (Relative *g*_s_) | SD Group | Genotype MUNQ | Genotype Name | Replicate | Location |
| --- | --- | --- | --- | --- | --- | --- | --- | --- | --- | --- |
| 0.33 | 0.22 | 10 | 100 | 67 | 10 | HSD | 1478 | Priscilla | A | CHE |
| 0.38 | 0.23 | 18 | 100 | 61 | 18 | HSD | 1478 | Priscilla | A | ESP |
| 0.37 | 0.23 | 10 | 100 | 70 | 16 | HSD | 1478 | Priscilla | A | FRA |
| 0.23 | 0.1 | 16 | 100 | 48 | 19 | HSD | 1478 | Priscilla | A | ITA |
| 0.29 | 0.23 | 5 | 91 | 72 | 5 | LSD | 472 | Rouget | A | CHE |
| 0.23 | 0.14 | 21 | 100 | 61 | 21 | LSD | 472 | Rouget | A | ESP |
| 0.25 | 0.16 | 18 | 96 | 62 | 18 | LSD | 472 | Rouget | A | FRA |
| 0.21 | 0.03 | 13 | 100 | 19 | 21 | LSD | 472 | Rouget | A | ITA |
| 0.31 | 0.16 | 8 | 97 | 50 | 9 | MSD-Comm | 739 | Gala | A | CHE |
| 0.26 | 0.11 | 13 | 96 | 41 | 13 | MSD-Comm | 739 | Gala | A | ESP |
| 0.31 | 0.13 | 28 | 94 | 39 | 28 | MSD-Comm | 739 | Gala | A | FRA |
| 0.28 | 0.15 | 3 | 100 | 60 | 16 | MSD-Comm | 739 | Gala | A | ITA |
| 0.49 | 0.24 | 8 | 100 | 63 | 19 | HSD | 1478 | Priscilla | B | CHE |
| 0.41 | 0.32 | 20 | 93 | 73 | 20 | HSD | 1478 | Priscilla | B | ESP |
| 0.31 | 0.23 | 9 | 100 | 85 | 20 | HSD | 1478 | Priscilla | B | FRA |
| 0.2 | 0.12 | 3 | 100 | 67 | 20 | HSD | 1478 | Priscilla | B | ITA |
| 0.31 | 0.22 | 19 | 99 | 71 | 19 | LSD | 472 | Rouget | B | CHE |
| 0.27 | 0.19 | 12 | 96 | 68 | 12 | LSD | 472 | Rouget | B | ESP |
| 0.2 | 0.11 | 11 | 95 | 52 | 11 | LSD | 472 | Rouget | B | FRA |
| 0.13 | 0.08 | 9 | 93 | 57 | 9 | LSD | 472 | Rouget | B | ITA |
| 0.34 | 0.25 | 15 | 100 | 81 | 24 | MSD-Comm | 739 | Gala | B | CHE |
| 0.36 | 0.21 | 20 | 100 | 68 | 22 | MSD-Comm | 739 | Gala | B | ESP |
| 0.35 | 0.15 | 3 | 90 | 38 | 3 | MSD-Comm | 739 | Gala | B | FRA |
| 0.27 | 0.17 | 11 | 100 | 65 | 17 | MSD-Comm | 739 | Gala | B | ITA |
| 0.38 | 0.27 | 18 | 100 | 73 | 18 | HSD | 1478 | Priscilla | C | CHE |
| 0.44 | 0.25 | 16 | 100 | 57 | 16 | HSD | 1478 | Priscilla | C | ESP |
| 0.32 | 0.21 | 3 | 100 | 66 | 3 | HSD | 1478 | Priscilla | C | FRA |
| 0.24 | 0.14 | 14 | 92 | 54 | 14 | HSD | 1478 | Priscilla | C | ITA |
| 0.3 | 0.22 | 13 | 97 | 71 | 13 | LSD | 472 | Rouget | C | CHE |
| 0.29 | 0.2 | 14 | 100 | 77 | 18 | LSD | 472 | Rouget | C | ESP |
| 0.27 | 0.18 | 20 | 93 | 62 | 20 | LSD | 472 | Rouget | C | FRA |
| 0.11 | 0.04 | 14 | 79 | 29 | 14 | LSD | 472 | Rouget | C | ITA |
| 0.33 | 0.18 | 17 | 100 | 55 | 17 | MSD-Comm | 739 | Gala | C | CHE |
| 0.37 | 0.22 | 15 | 100 | 65 | 15 | MSD-Comm | 739 | Gala | C | ESP |
| 0.37 | 0.25 | 16 | 100 | 76 | 13 | MSD-Comm | 739 | Gala | C | FRA |
| 0.27 | 0.12 | 16 | 96 | 43 | 16 | MSD-Comm | 739 | Gala | C | ITA |

**Table S3:** Stomatal conductance (*g_s_*) kinetics parameters extracted from the sigmoidal equation (Eqn 3) when light was increased back again to 1500 PAR for 30 minutes (recovery phase) for each replicate of each studied genotype in each location Spain (ESP), France (FRA), Italy (ITA), Switzerland (CHE).

| $\boldsymbol{G}_{\boldsymbol{max}}$ (*g*_s_) | $\boldsymbol{G}_{\boldsymbol{min}}$ (*g*_s_) | $\boldsymbol{\lambda}$  (*g*_s_) | $\boldsymbol{k}_{\boldsymbol{i}}$  (*g*_s_) | $\boldsymbol{Sl}_{\boldsymbol{max}}$ (*g*_s_) | $\boldsymbol{\tau}_{\boldsymbol{i}}$  (*g*_s_) | $\boldsymbol{G}_{\boldsymbol{max}}$ (Relative *g*_s_) | $\boldsymbol{G}_{\boldsymbol{min}}$ (Relative *g*_s_) | $\boldsymbol{\lambda}$  (Relative *g*_s_) | $\boldsymbol{k}_{\boldsymbol{i}}$  (Relative *g*_s_) | $\boldsymbol{Sl}_{\boldsymbol{max}}$  (Relative *g*_s_) | $\boldsymbol{\tau}_{\boldsymbol{i}}$  (Relative *g*_s_) | SD Group | Genotype MUNQ | Genotype name | | Replicate | Location |
| --- | --- | --- | --- | --- | --- | --- | --- | --- | --- | --- | --- | --- | --- | --- | --- | --- | --- |
| 0.33 | 0.25 | 2 | 9.97 | 0.29 | 7 | 100 | 76 | 2 | 9.97 | 0.88 | 7 | HSD | 1478 | Priscilla | A | | CHE |
| 0.38 | 0.22 | 3 | 11.22 | 0.66 | 9 | 100 | 58 | 3 | 11.60 | 1.79 | 9 | HSD | 1478 | Priscilla | A | | ESP |
| 0.37 | 0.19 | 3 | 83.13 | 5.50 | 47 | 100 | 58 | 3 | 24.84 | 3.84 | 16 | HSD | 1478 | Priscilla | A | | FRA |
| 0.23 | 0.1 | 2 | 14.32 | 0.68 | 10 | 100 | 48 | 2 | 13.09 | 2.50 | 9 | HSD | 1478 | Priscilla | A | | ITA |
| 0.29 | 0.23 | 3 | 11.39 | 0.25 | 9 | 91 | 72 | 3 | 10.96 | 0.77 | 9 | LSD | 472 | Rouget | A | | CHE |
| 0.23 | 0.15 | 3 | 8.29 | 0.24 | 7 | 100 | 65 | 3 | 8.40 | 1.08 | 7 | LSD | 472 | Rouget | A | | ESP |
| 0.25 | 0.18 | 3 | 14.04 | 0.36 | 10 | 96 | 69 | 3 | 12.53 | 1.24 | 10 | LSD | 472 | Rouget | A | | FRA |
| 0.21 | 0.02 | 3 | 13.57 | 0.95 | 10 | 100 | 12 | 3 | 9.19 | 2.97 | 8 | LSD | 472 | Rouget | A | | ITA |
| 0.31 | 0.18 | 12 | 8.13 | 0.39 | 16 | 97 | 56 | 12 | 8.25 | 1.24 | 16 | MSD-Comm | 739 | Gala | A | | CHE |
| 0.26 | 0.11 | 5 | 15.22 | 0.84 | 13 | 96 | 41 | 5 | 15.06 | 3.05 | 13 | MSD-Comm | 739 | Gala | A | | ESP |
| 0.31 | 0.18 | 3 | 47.27 | 2.26 | 28 | 94 | 55 | 3 | 44.83 | 6.43 | 27 | MSD-Comm | 739 | Gala | A | | FRA |
| 0.28 | 0.14 | 4 | 113.05 | 5.82 | 64 | 100 | 56 | 4 | 107.53 | 17.41 | 61 | MSD-Comm | 739 | Gala | A | | ITA |
| 0.49 | 0.28 | 17 | 9.67 | 0.75 | 22 | 100 | 74 | 15 | 5.34 | 0.51 | 18 | HSD | 1478 | Priscilla | B | | CHE |
| 0.41 | 0.36 | 3 | 16.49 | 0.30 | 12 | 93 | 82 | 3 | 15.78 | 0.64 | 11 | HSD | 1478 | Priscilla | B | | ESP |
| 0.31 | 0.22 | 3 | 28.78 | 0.95 | 18 | 100 | 81 | 3 | 14.20 | 0.99 | 11 | HSD | 1478 | Priscilla | B | | FRA |
| 0.2 | 0.09 | 2 | 34.65 | 1.40 | 20 | 100 | 50 | 2 | 21.10 | 3.88 | 13 | HSD | 1478 | Priscilla | B | | ITA |
| 0.31 | 0.25 | 2 | 9.66 | 0.21 | 7 | 99 | 81 | 2 | 8.53 | 0.56 | 7 | LSD | 472 | Rouget | B | | CHE |
| 0.27 | 0.19 | 3 | 12.86 | 0.38 | 10 | 96 | 68 | 3 | 12.84 | 1.32 | 10 | LSD | 472 | Rouget | B | | ESP |
| 0.2 | 0.11 | 3 | 3.78 | 0.13 | 5 | 95 | 52 | 3 | 3.77 | 0.60 | 5 | LSD | 472 | Rouget | B | | FRA |
| 0.13 | 0.07 | 2 | 6.21 | 0.14 | 5 | 93 | 50 | 2 | 6.10 | 0.96 | 5 | LSD | 472 | Rouget | B | | ITA |
| 0.34 | 0.26 | 2 | 16.39 | 0.48 | 11 | 100 | 84 | 2 | 12.05 | 0.71 | 8 | MSD-Comm | 739 | Gala | B | | CHE |
| 0.36 | 0.28 | 3 | 175.61 | 5.17 | 96 | 100 | 90 | 3 | 70.39 | 2.59 | 40 | MSD-Comm | 739 | Gala | B | | ESP |
| 0.35 | 0.27 | 2 | 11.35 | 0.33 | 8 | 80 | 61 | 2 | 11.71 | 0.82 | 8 | MSD-Comm | 739 | Gala | B | | FRA |
| 0.27 | 0.17 | 2 | 30.33 | 1.12 | 18 | 100 | 65 | 2 | 28.22 | 3.63 | 17 | MSD-Comm | 739 | Gala | B | | ITA |
| 0.38 | 0.29 | 3 | 13.19 | 0.44 | 10 | 100 | 78 | 3 | 12.39 | 1.00 | 10 | HSD | 1478 | Priscilla | C | | CHE |
| 0.44 | 0.27 | 9 | 13.30 | 0.83 | 16 | 100 | 61 | 9 | 13.25 | 1.90 | 16 | HSD | 1478 | Priscilla | C | | ESP |
| 0.32 | 0.21 | 4 | 11.53 | 0.47 | 10 | 100 | 66 | 4 | 11.50 | 1.44 | 10 | HSD | 1478 | Priscilla | C | | FRA |
| 0.24 | 0.12 | 2 | 19.89 | 0.88 | 13 | 92 | 46 | 2 | 20.19 | 3.42 | 13 | HSD | 1478 | Priscilla | C | | ITA |
| 0.3 | 0.26 | 2 | 4.25 | 0.06 | 4 | 97 | 84 | 2 | 4.14 | 0.20 | 4 | LSD | 472 | Rouget | C | | CHE |
| 0.29 | 0.21 | 2 | 18.22 | 0.54 | 12 | 100 | 81 | 2 | 15.91 | 1.11 | 10 | LSD | 472 | Rouget | C | | ESP |
| 0.27 | 0.21 | 3 | 9.08 | 0.20 | 8 | 93 | 72 | 3 | 8.98 | 0.69 | 8 | LSD | 472 | Rouget | C | | FRA |
| 0.11 | 0.02 | 3 | 2.77 | 0.09 | 4 | 79 | 14 | 3 | 2.83 | 0.68 | 5 | LSD | 472 | Rouget | C | | ITA |
| 0.33 | 0.19 | 4 | 16.29 | 0.84 | 13 | 100 | 58 | 4 | 16.29 | 2.52 | 13 | MSD-Comm | 739 | Gala | C | | CHE |
| 0.37 | 0.23 | 5 | 15.45 | 0.80 | 13 | 100 | 68 | 5 | 12.66 | 1.49 | 12 | MSD-Comm | 739 | Gala | C | | ESP |
| 0.37 | 0.3 | 2 | 15.02 | 0.88 | 12 | 100 | 91 | 0 | 13.87 | 0.45 | 9 | MSD-Comm | 739 | Gala | C | | FRA |
| 0.27 | 0.1 | 4 | 14.07 | 0.88 | 11 | 96 | 36 | 4 | 13.87 | 3.06 | 11 | MSD-Comm | 739 | Gala | C | | ITA |

**Table S4:** Net carbon assimilation (*A_net_*) time constant ($\tau_{a}$) to reach 95% of the steady-state *A_net_* when light was decreased from 1500 PAR to 100 PAR for a set period of 30 minutes (response phase) for each replicate of each studied genotype in each location Spain (ESP), France (FRA), Italy (ITA), Switzerland (CHE).

| $\boldsymbol{\tau}_{\boldsymbol{a}}$  (*A_net_*) | $\boldsymbol{\tau}_{\boldsymbol{a}}$  (Relative *A_net_*) | Genotype MUNQ | Genotype Name | SD Group | Replicate | Location |
| --- | --- | --- | --- | --- | --- | --- |
| 18 | 22 | 1478 | Priscilla | HSD | A | CHE |
| 22 | 22 | 1478 | Priscilla | HSD | A | ESP |
| 20 | 31 | 1478 | Priscilla | HSD | A | FRA |
| 37 | 37 | 1478 | Priscilla | HSD | A | ITA |
| 17 | 26 | 472 | Rouget | LSD | A | CHE |
| 28 | 35 | 472 | Rouget | LSD | A | ESP |
| 23 | 21 | 472 | Rouget | LSD | A | FRA |
| 37 | 37 | 472 | Rouget | LSD | A | ITA |
| 16 | 19 | 739 | Gala | MSD-Comm | A | CHE |
| 37 | 37 | 739 | Gala | MSD-Comm | A | ESP |
| 37 | 37 | 739 | Gala | MSD-Comm | A | FRA |
| 15 | 15 | 739 | Gala | MSD-Comm | A | ITA |
| 11 | 15 | 1478 | Priscilla | HSD | B | CHE |
| 16 | 13 | 1478 | Priscilla | HSD | B | ESP |
| 37 | 37 | 1478 | Priscilla | HSD | B | FRA |
| 18 | 17 | 1478 | Priscilla | HSD | B | ITA |
| 37 | 37 | 472 | Rouget | LSD | B | CHE |
| 30 | 30 | 472 | Rouget | LSD | B | ESP |
| 37 | 37 | 472 | Rouget | LSD | B | FRA |
| 15 | 16 | 472 | Rouget | LSD | B | ITA |
| 15 | 13 | 739 | Gala | MSD-Comm | B | CHE |
| 37 | 37 | 739 | Gala | MSD-Comm | B | ESP |
| 34 | 34 | 739 | Gala | MSD-Comm | B | FRA |
| 15 | 13 | 739 | Gala | MSD-Comm | B | ITA |
| 19 | 28 | 1478 | Priscilla | HSD | C | CHE |
| 16 | 16 | 1478 | Priscilla | HSD | C | ESP |
| 18 | 28 | 1478 | Priscilla | HSD | C | FRA |
| 16 | 14 | 1478 | Priscilla | HSD | C | ITA |
| 17 | 17 | 472 | Rouget | LSD | C | CHE |
| 37 | 37 | 472 | Rouget | LSD | C | ESP |
| 37 | 37 | 472 | Rouget | LSD | C | FRA |
| 13 | 12 | 472 | Rouget | LSD | C | ITA |
| 16 | 17 | 739 | Gala | MSD-Comm | C | CHE |
| 18 | 18 | 739 | Gala | MSD-Comm | C | ESP |
| 18 | 18 | 739 | Gala | MSD-Comm | C | FRA |
| 13 | 14 | 739 | Gala | MSD-Comm | C | ITA |

**Table S5:** Net carbon assimilation (*A_net_*) time constant ($\tau_{a}$) to reach 95% of the steady-state *A_net_* when light was increased from 100 PAR to 1500 PAR for a set period of 30 minutes (recovery phase) for each replicate of each studied genotype in each location Spain (ESP), France (FRA), Italy (ITA), Switzerland (CHE).

| $\boldsymbol{\tau}_{\boldsymbol{a}}$  (*A_net_*) | $\boldsymbol{\tau}_{\boldsymbol{a}}$  (Relative *A_net_*) | Genotype MUNQ | Genotype Name | SD Group | Replicate | Location |
| --- | --- | --- | --- | --- | --- | --- |
| 53 | 54 | 1478 | Priscilla | HSD | A | CHE |
| 38 | 38 | 1478 | Priscilla | HSD | A | ESP |
| 56 | 56 | 1478 | Priscilla | HSD | A | FRA |
| 68 | 66 | 1478 | Priscilla | HSD | A | ITA |
| 54 | 52 | 472 | Rouget | LSD | A | CHE |
| 54 | 57 | 472 | Rouget | LSD | A | ESP |
| 57 | 57 | 472 | Rouget | LSD | A | FRA |
| 68 | 68 | 472 | Rouget | LSD | A | ITA |
| 65 | 65 | 739 | Gala | MSD-Comm | A | CHE |
| 62 | 65 | 739 | Gala | MSD-Comm | A | ESP |
| 46 | 46 | 739 | Gala | MSD-Comm | A | FRA |
| 51 | 51 | 739 | Gala | MSD-Comm | A | ITA |
| 59 | 59 | 1478 | Priscilla | HSD | B | CHE |
| 46 | 46 | 1478 | Priscilla | HSD | B | ESP |
| 47 | 48 | 1478 | Priscilla | HSD | B | FRA |
| 58 | 57 | 1478 | Priscilla | HSD | B | ITA |
| 49 | 50 | 472 | Rouget | LSD | B | CHE |
| 56 | 58 | 472 | Rouget | LSD | B | ESP |
| 52 | 53 | 472 | Rouget | LSD | B | FRA |
| 68 | 67 | 472 | Rouget | LSD | B | ITA |
| 45 | 45 | 739 | Gala | MSD-Comm | B | CHE |
| 43 | 44 | 739 | Gala | MSD-Comm | B | ESP |
| 48 | 48 | 739 | Gala | MSD-Comm | B | FRA |
| 51 | 50 | 739 | Gala | MSD-Comm | B | ITA |
| 49 | 50 | 1478 | Priscilla | HSD | C | CHE |
| 50 | 51 | 1478 | Priscilla | HSD | C | ESP |
| 59 | 61 | 1478 | Priscilla | HSD | C | FRA |
| 58 | 58 | 1478 | Priscilla | HSD | C | ITA |
| 57 | 56 | 472 | Rouget | LSD | C | CHE |
| 48 | 49 | 472 | Rouget | LSD | C | ESP |
| 53 | 54 | 472 | Rouget | LSD | C | FRA |
| 61 | 60 | 472 | Rouget | LSD | C | ITA |
| 55 | 56 | 739 | Gala | MSD-Comm | C | CHE |
| 57 | 57 | 739 | Gala | MSD-Comm | C | ESP |
| 45 | 45 | 739 | Gala | MSD-Comm | C | FRA |
| 52 | 52 | 739 | Gala | MSD-Comm | C | ITA |


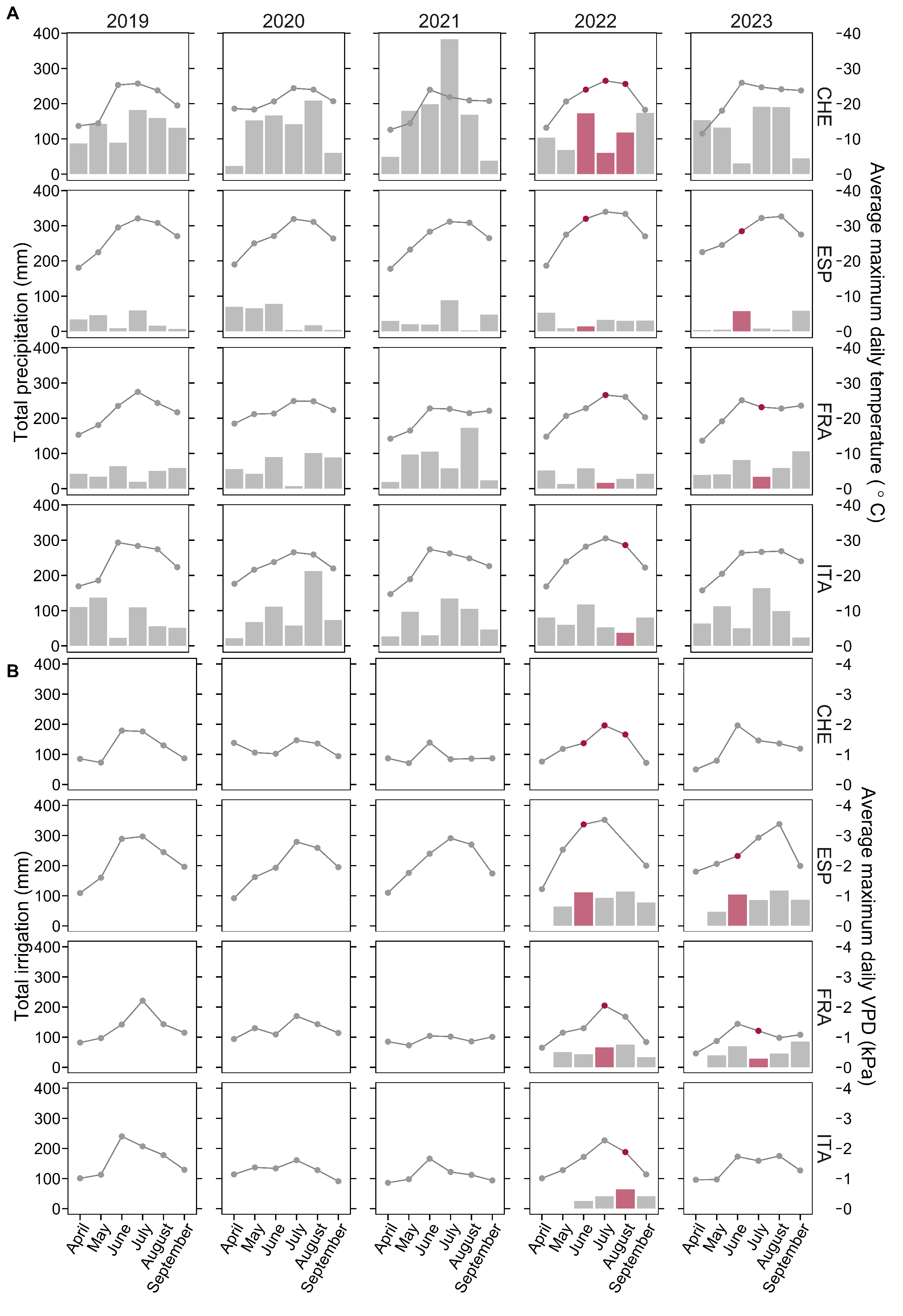


**Figure S1: Growing season conditions in the apple REFPOP orchard in Spain (ESP), France (FRA), Italy (ITA), Switzerland (CHE) from 2019 to 2023.** Lines indicate (A) the average maximum daily temperature (right y-axis) and (B) the average maximum daily vapor pressure deficit (VPD). These variables were calculated by extracting the daily maximum value between 12:00 and 14:00 and averaging across the month. Bars in (A) indicate the cumulative monthly precipitation and (B) the supplied irrigation (left y-axis). ESP, FRA, and ITA applied irrigation, CHE did not. Irrigation data were available for ESP, FRA, and ITA in 2022, and for ESP and FRA in 2023. Red-highlighted bars in 2022 and in 2023 indicate the months during which the leaf physiological measurements sampling was conducted at each specific location.


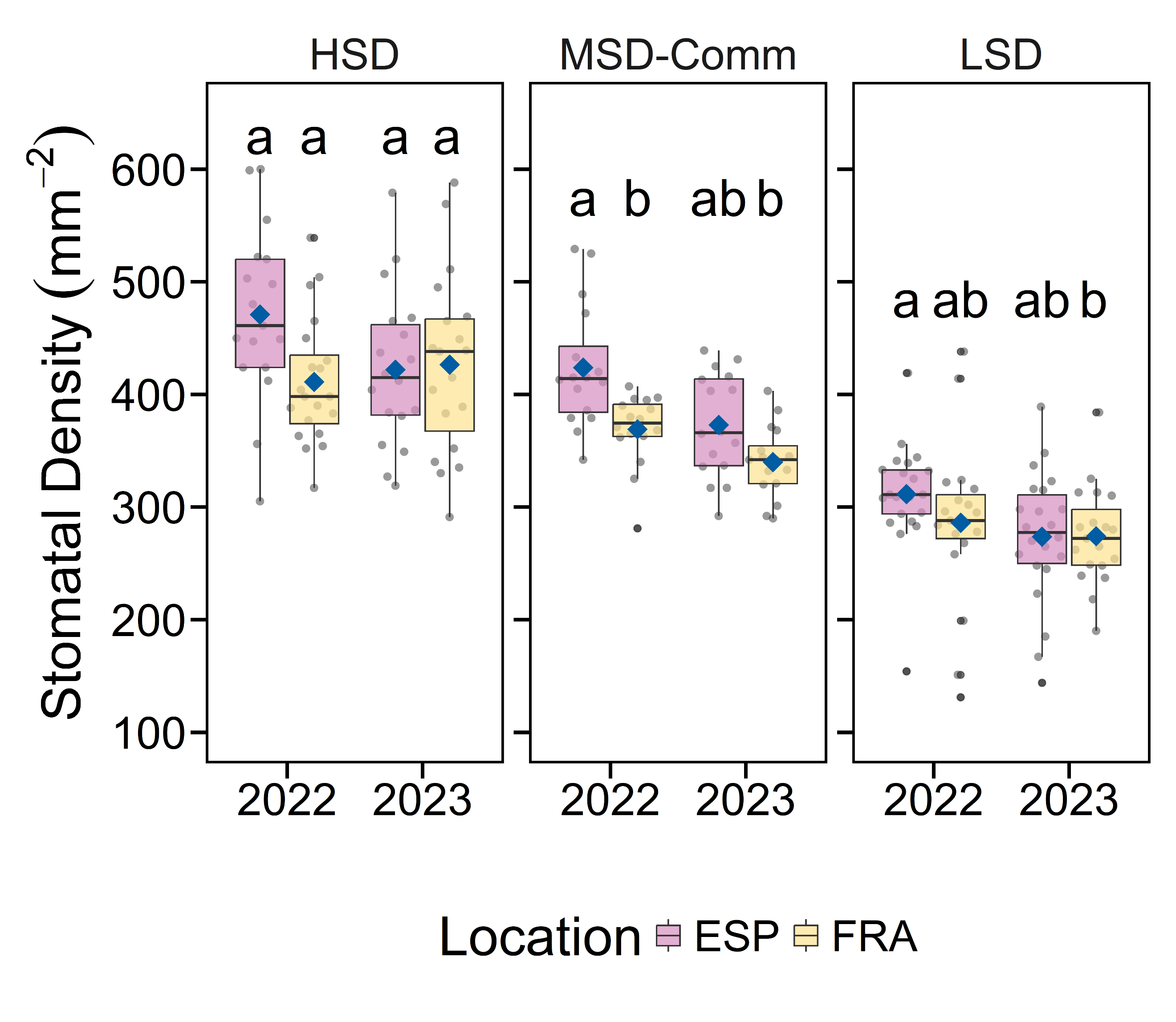


**Figure S2: Stomatal density (SD) remains consistent across locations and across years.** Boxplots with means (blue diamonds) and individual tree data points of SD for the selected subsets of accessions in three SD groups (*n* = 110): high SD (HSD), commercial cultivars with medium SD (MSD-Comm) and low SD (LSD) in 2022 and in 2023 across two locations: Spain (ESP, pink colored) and France (FRA, yellow colored). Significant differences across years and locations within each SD group were assessed using Wilcoxon tests and indicated with different letters (*p* < 0.05, *p*-values were adjusted with the Bonferroni correction). The box extends from the 1st quartile (25th percentile, lower edge) to the 3rd quartile (75th percentile, upper edge), with the median displayed as a line inside. Whiskers reach the smallest and largest values within 1.5 times the interquartile range, while outliers are shown as individual points beyond the whiskers.


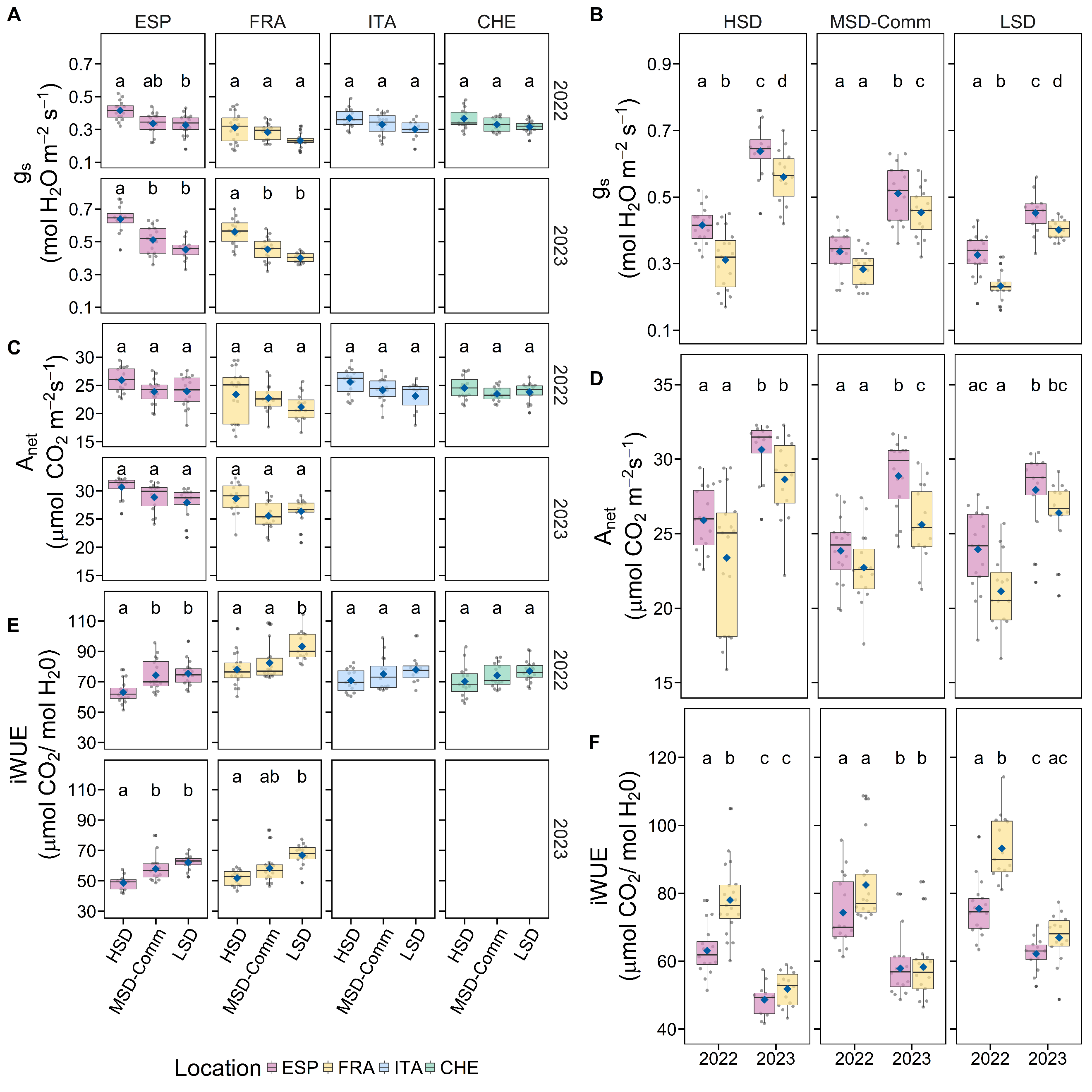


**Figure S3: Leaf gas-exchange measurements across stomatal density (SD) groups, locations and years**. Boxplots with means (blue diamonds) and individual tree data points of (A, B) stomatal conductance (*g_s_*), (C, D) net carbon assimilation (*A_net_*), (E, F) intrinsic water-use efficiency (iWUE) (*n* = 274). (A, C, E) across the SD groups: high SD (HSD), commercial cultivars with medium SD (MSD-Comm) and low SD (LSD); in the locations in Spain (ESP, pink coloured), France (FRA, yellow coloured), Italy (ITA, blue coloured), Switzerland (CHE, green coloured), in 2022 and 2023; (B, D, F) across years and locations within each SD group. The box extends from the 1^st^ quartile (25^th^ percentile, lower edge) to the 3^rd^ quartile (75^th^ percentile, upper edge), with the median displayed as a line inside. Whiskers reach the smallest and largest values within 1.5 times the interquartile range, while outliers are shown as individual points beyond the whiskers. Measurements from trees that were lacking fruit are omitted. Measurement conditions were set as follows: flow rate 600 μmol s^-1^; fan speed 10,000 rpm; [CO_2_] 415 ppm; PAR 1500 μmol photons m^-2^ s^-1^. Measurements were taken between 08:30 and 13:30, depending on the daily weather conditions. Significant differences across SD group within each location and year (A, C, E) and across years and locations within each SD group (B, D, F) were assessed using Wilcoxon tests and indicated with different letters (*p* < 0.05, *p*-values were adjusted with the Bonferroni correction).


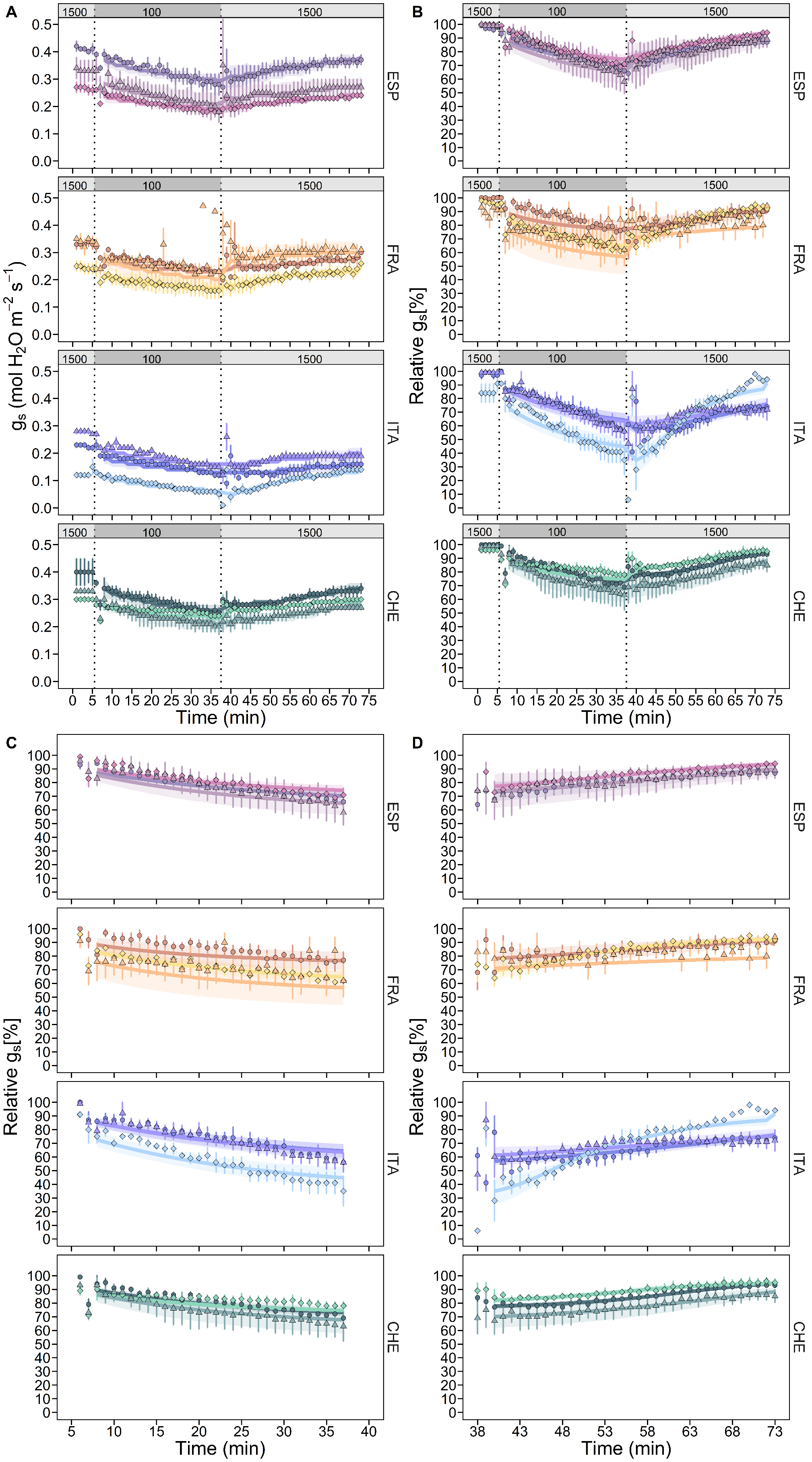


**Figure S4: Stomatal kinetics across locations.** Absolute response to changes in light intensity of (A) stomatal conductance (*g_s_*). Relative response to changes in light intensity of stomatal conductance (Relative *g_s_*) (B). Individual curves were normalized to the maximum value observed in each replicate. Response phase for the light transition from 1500 to 100 PAR of (C) Relative *g_s_*. Recovery phase for the light transition from 100 to 1500 PAR for (D) Relative *g_s_*. Points represent the mean value of three replications for each accession (*n* = 9) in each SD group: high SD (HSD) (represented by circles with dark colour shading), commercial cultivars with medium SD (MSD-Comm) (triangles with medium colour shading) and low SD (LSD) (diamonds with light color shading) in the locations in Spain (ESP, pink coloured), France (FRA, yellow coloured), Italy (ITA, blue coloured), Switzerland (CHE, green coloured). Vertical error bars represent the standard error of the mean. Solid lines indicate the mean value of the temporal response model used to predict *g_s_* for each accession (*n* = 9) in each SD group and location. Line confidence intervals represent standard error of the predicted mean.


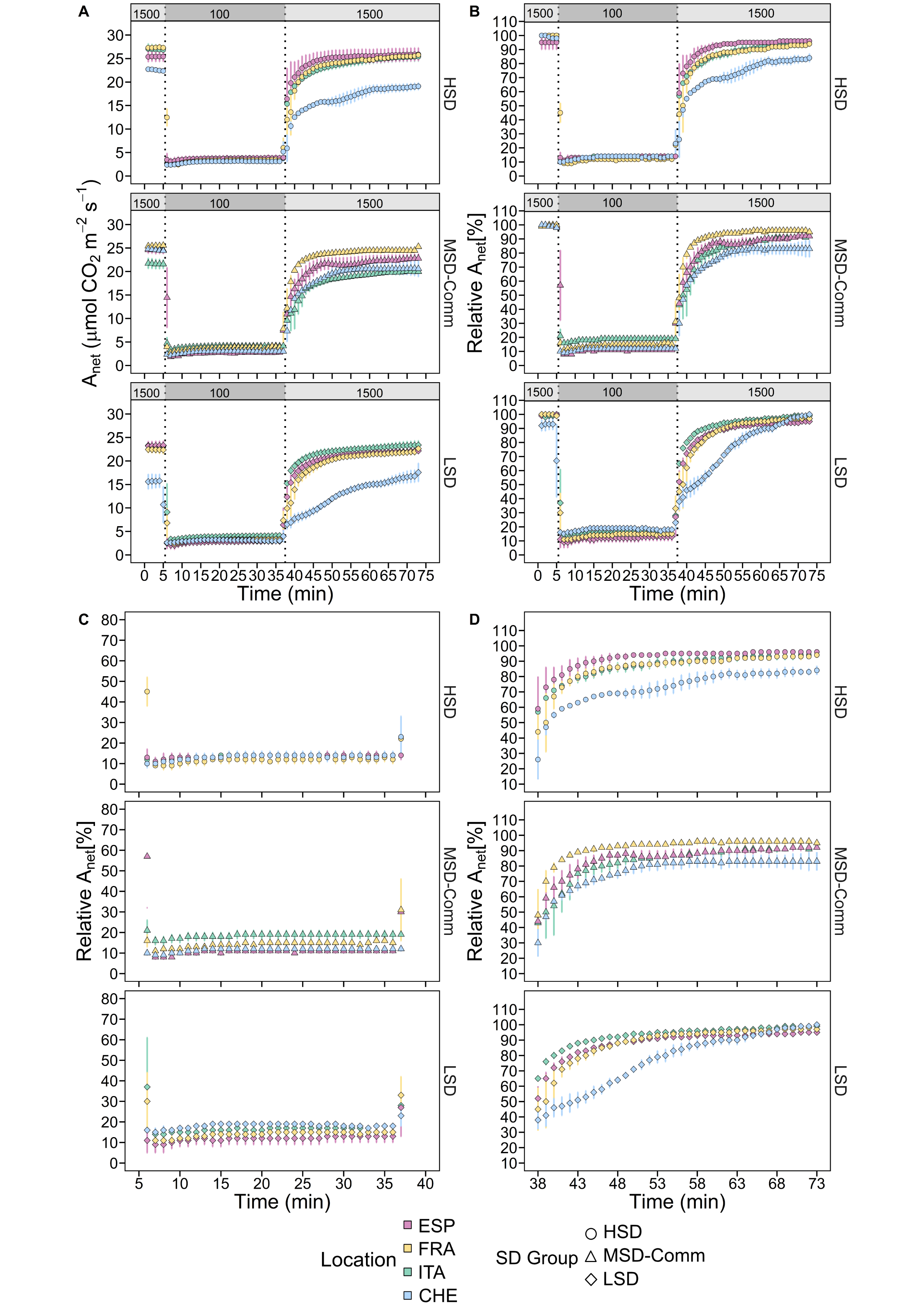


**Figure S5: Net carbon assimilation (*A_net_*) kinetics within stomatal density (SD) groups across locations.** Absolute response to changes in light intensity of (A) net carbon assimilation (*A_net_*). Relative response to changes in light intensity of (B) net carbon assimilation (Relative *A_net_*). Individual curves were normalized to the maximum value observed in each replicate. Response phase for the light transition from 1500 to 100 PAR of (C) Relative *A_net_*. Recovery phase for the light transition from 100 to 1500 PAR for (D) Relative *A_net_*. Points represent the mean value of three replications for each accession (*n* = 9) in the locations in Spain (ESP, pink coloured), France (FRA, yellow coloured), Italy (ITA, blue coloured), and Switzerland (CHE, green coloured). Vertical error bars represent standard error of the mean. Solid lines indicate the mean value of the temporal response model used to predict *g_s_* for each accession (*n* = 9) in each SD group and location. Line confidence intervals represent standard error of the predicted mean.


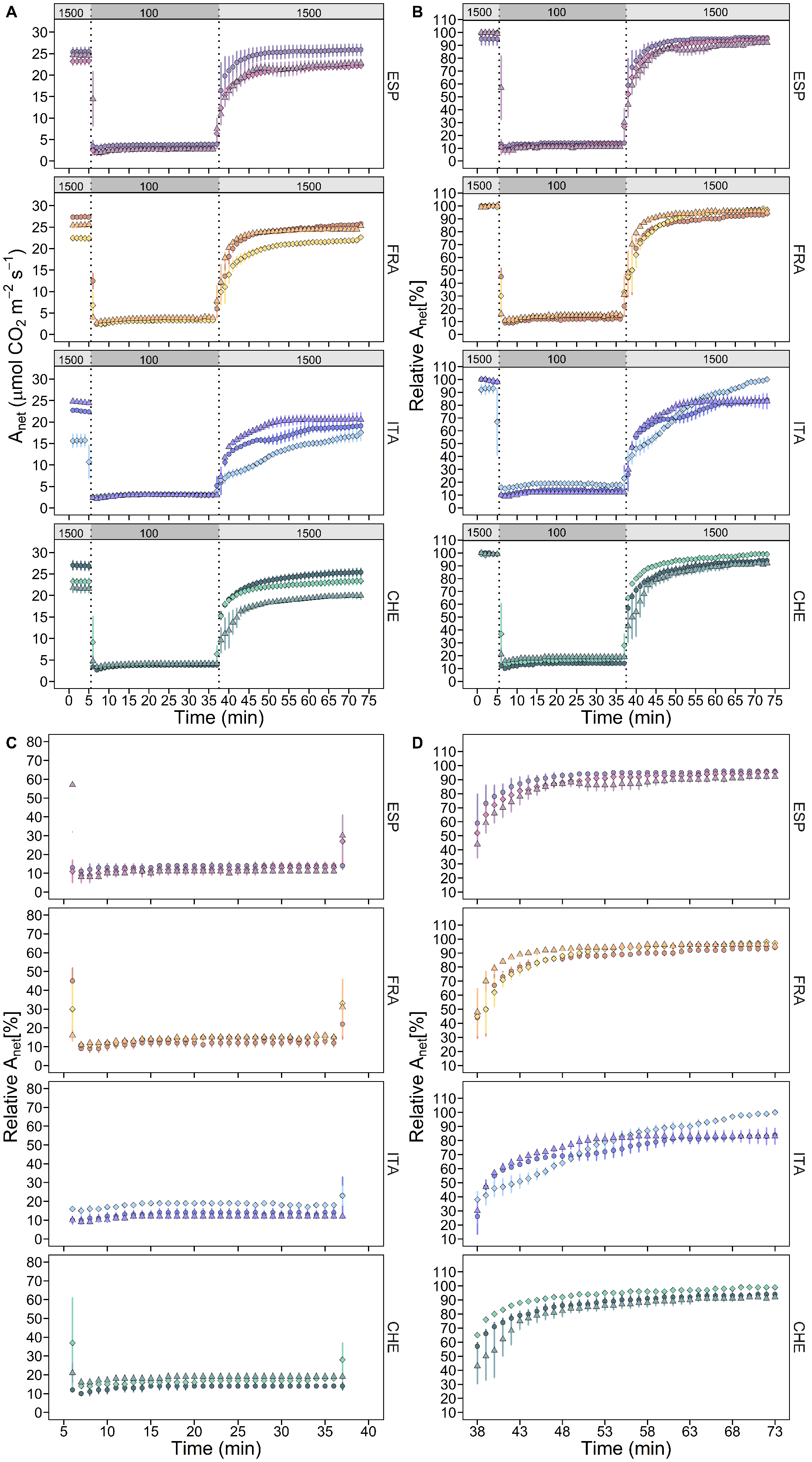


**Figure S6:** **Net carbon assimilation (*A_net_*) kinetics across locations.** Absolute response to changes in light intensity of (A) net carbon assimilation (*A_net_*). Relative response to changes in light intensity of (B) net carbon assimilation (Relative *A_net_*). Individual curves were normalized to the maximum value observed in each replicate. Response phase for the light transition from 1500 to 100 PAR of (C) Relative *A_net_*. Recovery phase for the light transition from 100 to 1500 PAR for (D) Relative *A_net_*. Points represent the mean value of three replications for each accession (*n* = 9) in each SD group: high SD (HSD) (represented by circles with dark colour shading), commercial cultivars with medium SD (MSD-Comm) (triangles with medium colour shading) and low SD (LSD) (diamonds with light colour shading) in each location in Spain (ESP, pink coloured), France (FRA, yellow coloured), Italy (ITA, blue coloured), and Switzerland (CHE, green coloured). Vertical error bars represent standard error of the mean. Solid lines indicate the mean value of the temporal response model used to predict *g_s_* for each accession (*n* = 9) in each SD group and location. Line confidence intervals represent standard error of the predicted mean.


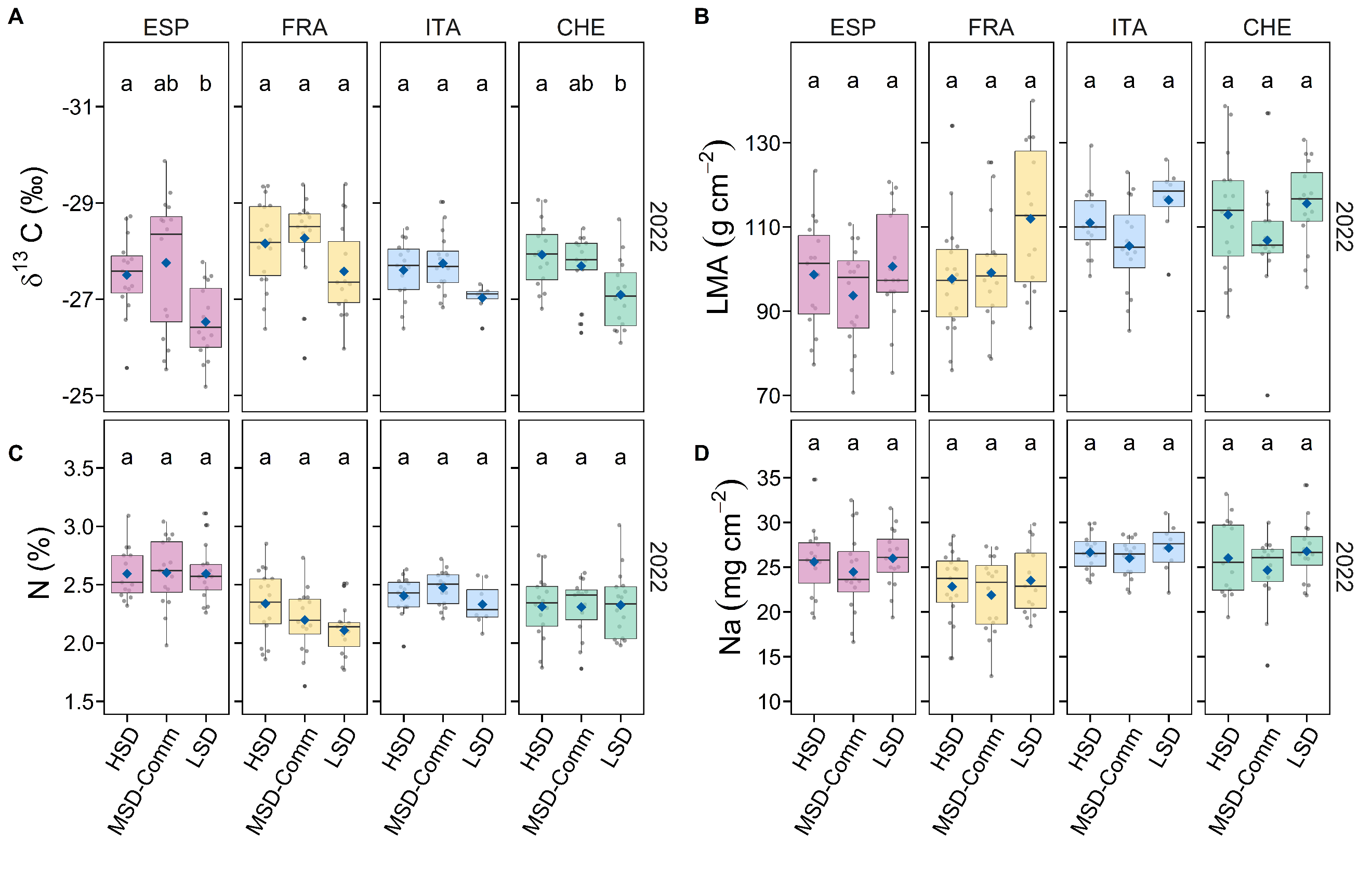


**Figure S7: Leaf traits across SD groups within each location.** Boxplots with means (blue diamonds) and individual tree data points of (A) integrated water-use efficiency (δ^13^C), (B) leaf mass area (LMA), (C) nitrogen percentage (%), (D) nitrogen content per unit leaf area (Na) within each location in Spain (ESP, pink coloured), France (FRA, yellow coloured), Italy (ITA, blue coloured), and Switzerland (CHE, green coloured) between the SD group (*n* = 189) : high SD (HSD), commercial cultivars with medium SD (MSD-Comm), and low SD (LSD). The box extends from the 1st quartile (25th percentile, lower edge) to the 3rd quartile (75th percentile, upper edge), with the median displayed as a line inside. Whiskers reach the smallest and largest values within 1.5 times the interquartile range, while outliers are shown as individual points beyond the whiskers. Measurements from trees that were lacking fruit are omitted. Significant differences across SD group within each location were assessed using Wilcoxon tests and indicated with different letters (*p* < 0.05, *p*-values were adjusted with the Bonferroni correction).


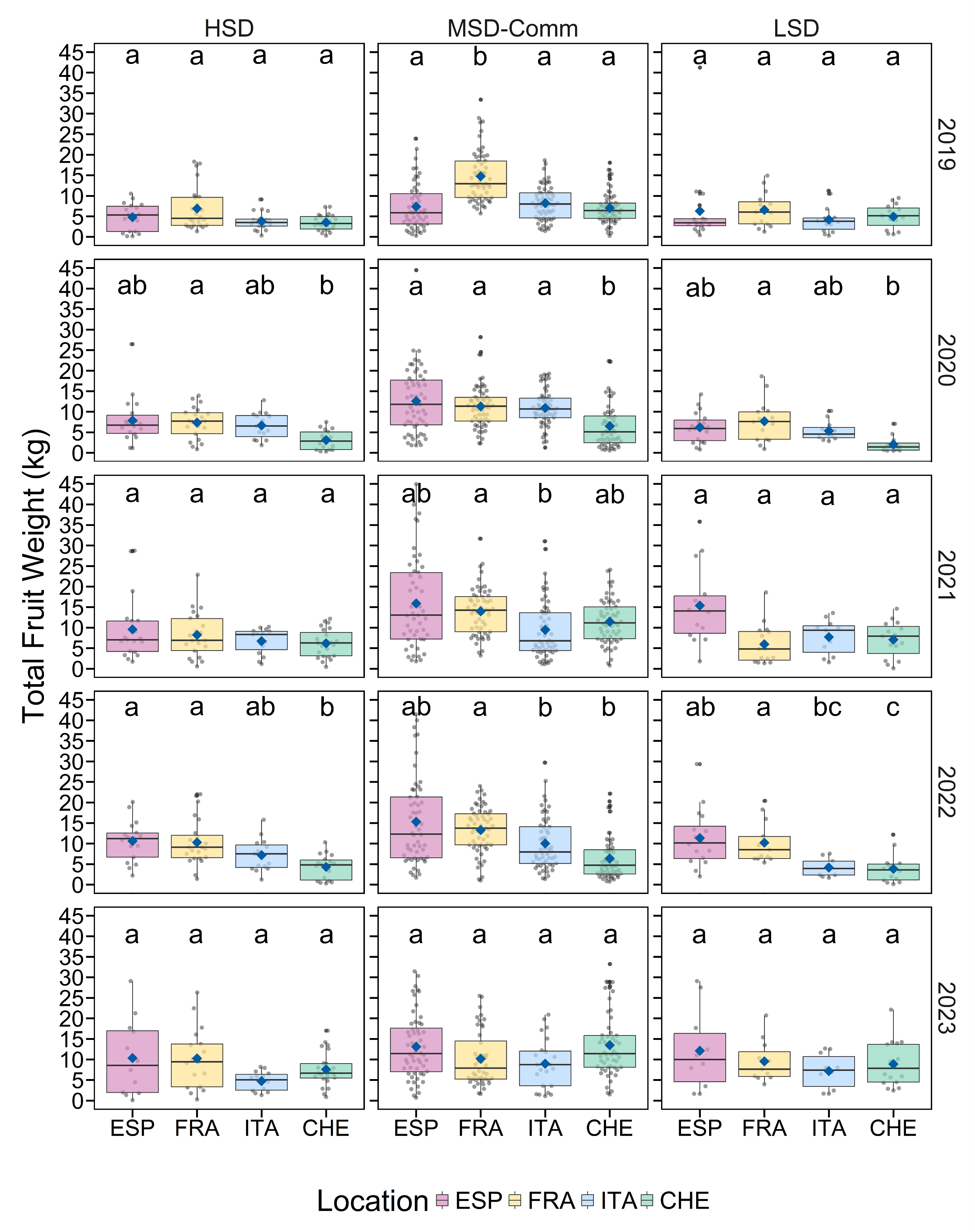


**Figure S8: Total Fruit Weight from 2019 to 2023 within stomatal density (SD) groups across locations.** Boxplots with means (blue diamonds) and individual tree data points of Total Fruit Weight (TFW) from 2019 to 2023 for each SD group (*n* = 1,702): high SD (HSD), commercial cultivars with medium SD (MSD-Comm) and low SD (LSD) in the locations in Spain (ESP, pink colored), France (FRA, yellow colored), Italy (ITA, blue colored), and Switzerland (CHE, green colored).The box extends from the 1st quartile (25th percentile, lower edge) to the 3rd quartile (75th percentile, upper edge), with the median displayed as a line inside. Whiskers reach the smallest and largest values within 1.5 times the interquartile range, while outliers are shown as individual points beyond the whiskers. Bearing and not bearing trees were included. Significant differences across locations within each SD group were assessed using Wilcoxon tests and indicated with different letters (*p* < 0.05, *p*-values were adjusted with the Bonferroni correction).


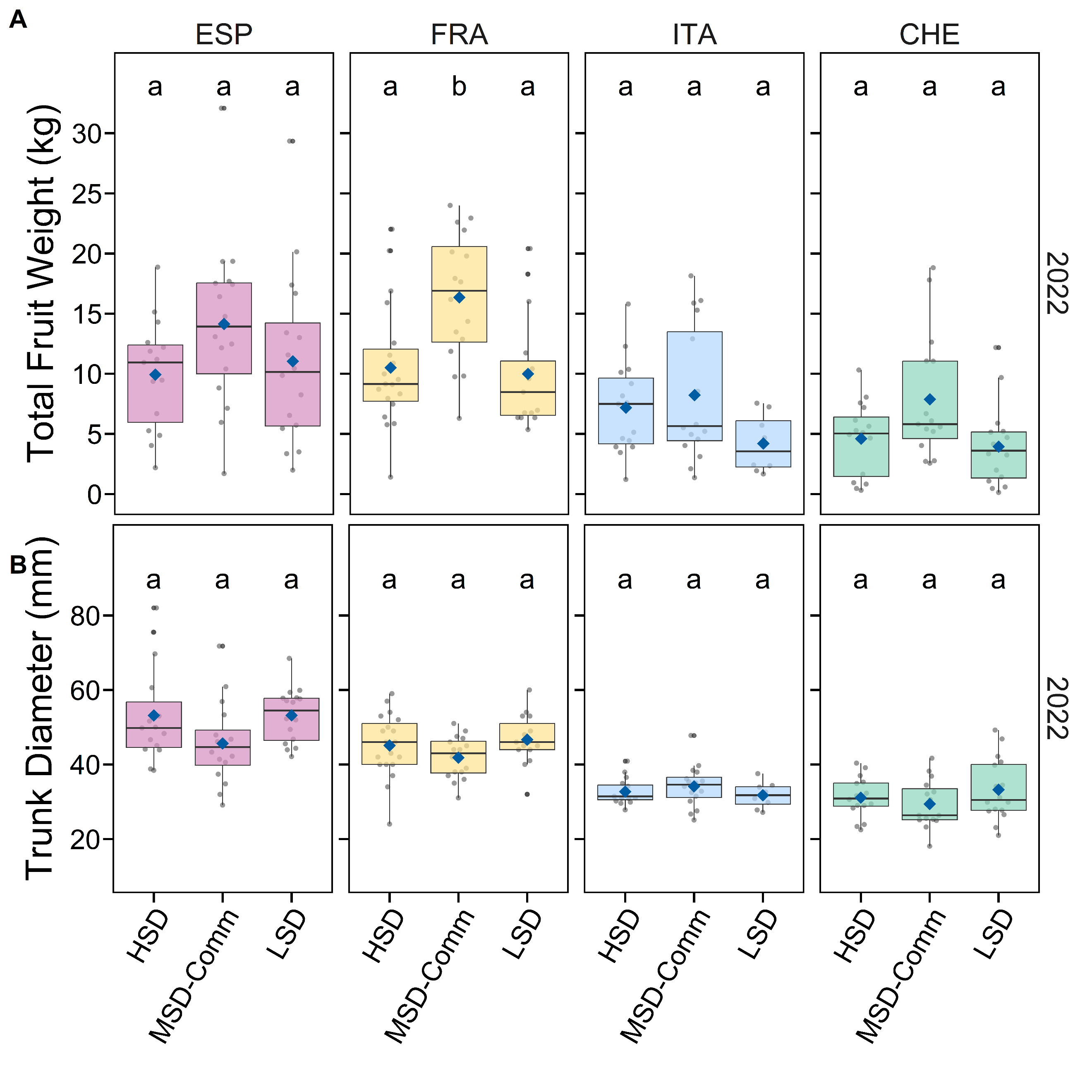


**Figure S9: Fruit yields and tree growth across SD groups within each location.** Boxplots with means (blue diamonds) and individual tree data points of (A) Total Fruit Weigh, (B) Trunk Diameter within each locations in Spain (ESP, pink coloured), France (FRA, yellow coloured), Italy (ITA, blue coloured), and Switzerland (CHE, green coloured) between the SD group (*n* = 189): high SD (HSD), commercial cultivars with medium SD (MSD-Comm), and low SD (LSD). The box extends from the 1st quartile (25th percentile, lower edge) to the 3rd quartile (75th percentile, upper edge), with the median displayed as a line inside. Whiskers reach the smallest and largest values within 1.5 times the interquartile range, while outliers are shown as individual points beyond the whiskers. Measurements from trees that were lacking fruit are omitted. Significant differences across SD group within each location were assessed using Wilcoxon tests and indicated with different letters (*p* < 0.05, *p*-values were adjusted with the Bonferroni correction).
